# A dynamic clamping approach using in silico IK1 current for discrimination of chamber-specific hiPSC-derived cardiomyocytes

**DOI:** 10.1038/s42003-023-04674-9

**Published:** 2023-03-18

**Authors:** Claudia Altomare, Chiara Bartolucci, Luca Sala, Carolina Balbi, Jacopo Burrello, Nicole Pietrogiovanna, Alessio Burrello, Sara Bolis, Stefano Panella, Martina Arici, Rolf Krause, Marcella Rocchetti, Stefano Severi, Lucio Barile

**Affiliations:** 1 Cardiovascular Theranostics, Istituto Cardiocentro Ticino, Ente Ospedaliero Cantonale, Lugano, Switzerland; 2 Laboratories for Translational Research, Ente Ospedaliero Cantonale, Bellinzona, Switzerland; 3 Euler institute, Università Svizzera italiana, Lugano, Switzerland; 4 Department of Electrical, Electronic and Information Engineering ‘Guglielmo Marconi’, University of Bologna, Cesena, Italy; 5 Istituto Auxologico Italiano IRCCS, Center for Cardiac Arrhythmias of Genetic Origin and Laboratory of Cardiovascular Genetics, Milan, Italy; 6 Department of Biotechnology and Biosciences, University of Milano-Bicocca, Milano, Italy; 7 Cellular and Molecular Cardiology, Istituto Cardiocentro Ticino, Ente Ospedaliero Cantonale, Lugano, Switzerland; 8 Center for Molecular Cardiology, University of Zurich, Zurich, Switzerland; 9 Division of Internal Medicine 4 and Hypertension Unit, Department of Medical Sciences, University of Torino, Torino, Italy; 10 Department of Electrical, Electronic and Information Engineering (DEI), University of Bologna, Bologna, Italy; 11 Faculty of Biomedical Sciences, Università Svizzera italiana, Lugano, Switzerland; 12 Institute of Life Science, Scuola Superiore Sant’Anna, Pisa, Italy

**Keywords:** Computational models, Cardiovascular biology

## Abstract

Human induced pluripotent stem cell (hiPSC)-derived cardiomyocytes (CM) constitute a mixed population of ventricular-, atrial-, nodal-like cells, limiting the reliability for studying chamber-specific disease mechanisms. Previous studies characterised CM phenotype based on action potential (AP) morphology, but the classification criteria were still undefined. Our aim was to use in silico models to develop an automated approach for discriminating the electrophysiological differences between hiPSC-CM. We propose the dynamic clamp (DC) technique with the injection of a specific I_K1_ current as a tool for deriving nine electrical biomarkers and blindly classifying differentiated CM. An unsupervised learning algorithm was applied to discriminate CM phenotypes and principal component analysis was used to visualise cell clustering. Pharmacological validation was performed by specific ion channel blocker and receptor agonist. The proposed approach improves the translational relevance of the hiPSC-CM model for studying mechanisms underlying inherited or acquired atrial arrhythmias in human CM, and for screening anti-arrhythmic agents.

## Abstract (web summary)

An in silico hybrid model to derive electrical biomarkers to be used as inputs for an unsupervised learning algorithm to classify differentiated hiPSC-derived cardiomyocytes from a mixed population of ventricular- and atrial-like cells.

## Introduction

Cardiomyocytes (CM) derived from human induced pluripotent stem cells (hiPSC) are mainly characterised by a heterogeneous mixture of immature ventricular-, atrial- and nodal-like CM phenotypes whose proportion depends on differentiation timing and protocols^1^. This aspect affects the identification and characterisation of electrical properties of hiPSC-CM subtypes, which is crucial in studies investigating chamber-specific disease mechanisms or addressing potential drug-induced cardiotoxicity during the development of pharmaceutical molecules^2^. Of particular concern is the evaluation of proarrhythmic effects with the use of antiarrhythmic drugs, in particular agents of class III that block potassium channel, prolong the QT interval and increase the risk for fatal arrythmia (torsades de pointes)^3,4^. For these reasons, US Food and Drug Administration (FDA) has mandated in vitro cardiotoxicity screening early in new drug development using hiPSC-CM (https://database.ich.org/sites/default/files/ICH_E14-S7B_QAs_Step2_2020_0827_0.pdf)^5^.

Nevertheless, antiarrhythmic drugs act through action potential (AP) shortening or prolonging effects, and the assessment of atrial- and ventricular-specific APs in human CM becomes critical. There are no molecular markers specific or selective for adult chamber-specific myocytes and attempts to determine the existence of distinct subpopulations of atrial and/or ventricular CM, during single cell patch-clamp recordings, mainly rely on qualitative morphological analyses of AP properties^2^. However, immature AP morphologies characterized by depolarized diastolic membrane potential (E_diast_) in standard hiPSC-CM culture makes difficult and largely debated the discrimination between atrial and ventricular AP phenotype^6–9^.

A valid and effective approach to overcome immature characteristics of hiPSC-CM is the injection of in silico inward rectifier K^+^ current (I_K1_) in loop with recorded cellular membrane potential in a real-time mode, namely dynamic clamp (DC) tool^10–14^. This technology allows the hyperpolarization of E_diast_ to values suitable for generating a mature AP waveform. However, applying a generic I_K1_ formulation on heterogeneous hiPSC-CM populations may result in a misleading interpretation of the cellular electrical response due to differences in the biophysical properties of ventricular *vs* atrial I_K1_; for instance, I_K1_ density and rectification are higher in human ventricles than in atria, thus leading to a chamber-specific contribution of I_K1_ to AP waveform^8,15^.

Here, we sought to empirically unravel hiPSC-CM chamber specification by using a machine learning approach to combine several electrical biomarkers recorded in DC mode based on state-of-the-art in silico I_K1_. We tested two I_K1_ formulations: (1) the one from O’Hara-Rudy (ORd) model of human ventricular AP^16^, which has been shown to perform well for induction of ventricular AP in hiPSC-CM but has never been validated on cells differentiated toward atrial phenotype^10,17^; (2) the one from the Koivumäki’s computational model of human atrial AP^18^, which has never been applied to hiPSC-CM in vitro. Electrical biomarkers obtained in DC mode recordings including APD_90_, APD_50_, and APD_20_ (representing AP duration measured at 90%, 50%, and 20% of the repolarisation phase respectively) were used to run an unsupervised learning algorithm to blindly classify the recorded CM. The specificity of the algorithm was pharmacologically validated testing the effect of 4-aminopyridine (4-AP) on AP, in that it’s the specific blocker of the atrial ultrarapid delayed rectifier potassium current (I_Kur_). The approach was then used to evaluate the differentiation efficiency of two protocols known to enrich for either atrial- or ventricular-like CM. Finally, we assessed the use of a Koivumäki I_K1_ formulation on atrial/ventricular cell types to determine how it impacts the interpretation of arrythmogenicity at the cellular level.

## Results

### Assessment of ventricular vs atrial markers

**Fig. 1:**
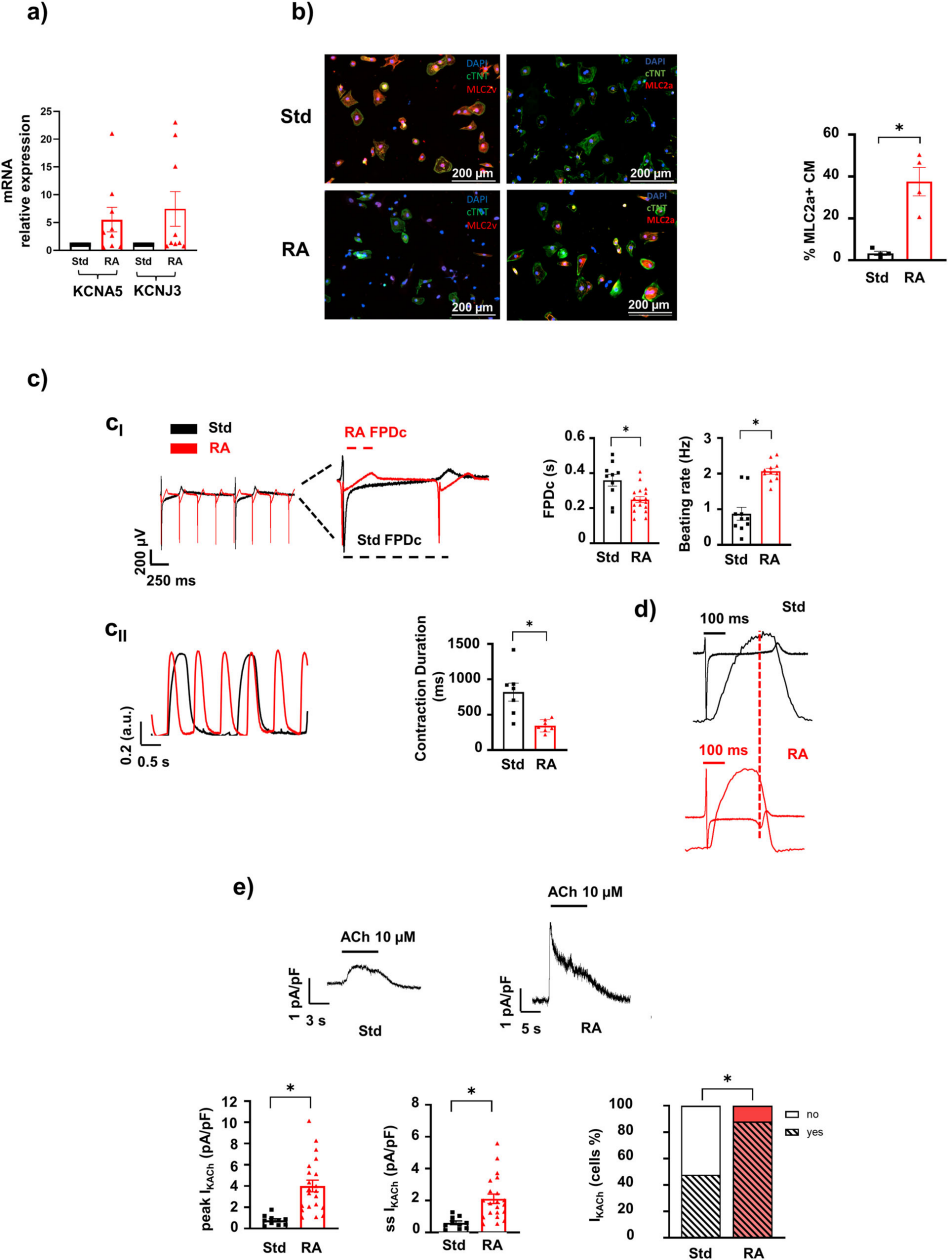
Molecular and functional assessment of atrial-like hiPSC-CM at 30 days of differentiation. **a** K^+^ channel KCNA5 and KCNJ3 mRNA relative expression in RA *vs* Std protocols (*n* = 9 technical replicates from 4 independent differentiations). **b** The percentage of atrial- *vs* ventricular-like cardiomyocytes (CM) was evaluated by immunofluorescence for the specific isoform of myosin light chain 2 (MLC2v, ventricular and MLC2a, atrial in red). Cells were counterstained for cardiac troponin T (TnT, in green) cells (*n* = 4 independent differentiations) scale bars = 200 μm. **c** Electrical (MEA, c_I_) and mechanical (MUSCLEMOTION,c_II_) recordings of RA-treated clusters of CMs compared to Std one. Higher beating rate and shorter FPDc was quantified from MEA (*n* = 10 and *n* = 17 clusters of Std and RA respectively, from 6 independent differentiations). Shorter contraction duration in mechanical RA events were analysed in MUSCLEMOTION recordings (*n* = 7 clusters of Std and RA, from 6 independent differentiations). **d** Overlapped recordings highlight different kinetic properties of excitation-contraction (EC) coupling in both conditions. Red dashed line showed electromechanical coupling in Std compared to RA cluster (**e**) Examples of ACh-elicited current recordings and analysis of peak steady-state, in single Std- (black) and RA-treated (red) CMs (*n* = 10 and *n* = 21, respectively from 2 independent differentiations). Percentage of ACh-responsive (yes) and not-responsive CMs (no) are quantified using both differentiation protocols. (Std standard protocol, RA retinoic acid, FPDc corrected field potential duration, Contr. ACh acetylcholine, I_KACh_ ACh-activated current). Data shown are mean ± SEM (*p < 0.05 paired *t*-test RA *vs* Std).

To obtain a consistent enrichment of the two main functional subtypes of hiPSC-CM (ventricular- and atrial-like CM), and use them as testing platform for the in silico models, we implemented previously published cardiac specific differentiation protocols: a standard protocol (Std), known to enrich the culture with ventricular-like CM^19^, and the retinoic acid (RA) protocol to induce atrial specification (protocol outline in Supplementary Fig. 1a)^20^. The presence of atrial-like CM was primarily confirmed at the mRNA level for the orphan nuclear transcription factors I and II (COUP-TF I/II), known to be the most significantly upregulated genes following RA treatment^21,22^. RA induced 16.81 ± 4.7 (*p* < 0.004)- and 10.4 ± 3.1 (*p* < 0.008)-fold increases in expression levels as compared to the Std protocol for COUP-TFI and COUP-TFII, respectively (Supplementary Fig. 1b). Similarly, mRNA levels of KCNA5 and KCNJ3 genes, encoding for atrial-specific potassium channels (K_V_1.5 and K_ir_3.1, respectively) downstream to COUP-TFs^20^, were upregulated following RA treatment (Fig. 1a). Moreover, immunofluorescence staining showed an increase in the proportion of hiPSC-CM expressing atrial myosin light chain 2 (MLC2a^+^) protein and a decrease in the proportion of cells expressing the ventricular isoform MLC2v, upon treatment with RA (Fig. 1b). Electrical (multielectrode arrays, MEA) and mechanical (MUSCLEMOTION) analyses of the clusters’ spontaneous activities, confirmed differential specification of Std *vs* RA protocol. We observed an increased beating rate in RA-treated cells (2.1 ± 0.1 Hz RA vs 0.9 ± 0.2 Hz Std) and a shortening of corrected field potential duration (FPDc) (0.25 ± 0.02 s RA *vs* 0.36 ± 0.03 s Std) measured by MEA that matched the reduction in the contraction duration (344 ± 32.6 ms RA vs 820 ± 127 ms Std) measured by MUSCLEMOTION (Fig. 1c). Examples of electromechanical coupling in the same cluster derived from Std (black traces) and RA (red traces) cultures are shown in Fig. 1d. All the other contraction parameters, time to peak, relaxation time, relaxation time at 90% and 50% of the amplitude (RT_90_ and RT_50_, respectively), showed a significant reduction in RA hiPSC-CM (Supplementary Fig. 1c–e, Supplementary Table 1). By voltage-clamp recordings on cluster-dissociated single cell, we tested the ability of differentiated CM to elicit acetylcholine (ACh)-dependent K^+^ current (I_KACh_)^20,23^. Such current is mediated by the KCNJ3 channel gene (Fig. 1e) and is responsible for the specific parasympathetic modulation of atrial tissue. The superfusion of high ACh concentration (10 µM) increased the percentage of I_KACh_-responsive CM from 47.8% in Std to 88% in RA conditions. Furthermore, the well-known short-term desensitisation process of the I_KACh_ well emerged in RA CMs only^24^. Taken together, these data clearly showed that the two differentiation protocols RA and Std, triggered the enrichment of CM towards an atrial- and ventricular-like phenotype respectively, however the efficiency of differentiation never reached a fully chamber specification. The attempt to distinguish different cell subtypes by assessing the presence of specific molecular biomarkers (e.g., different myosin isoforms and/or the presence of atrial-specific ACh-sensitive channel) failed to “uniquely assign” chamber-specific phenotype. We sought to achieve this specific goal by analysing multiple electrical parameters at single cell analysis, which is only feasible if a “physiological AP” is elicited by using DC mode.

### Optimisation of the critical I_K1_ conductance value

**Fig. 2:**
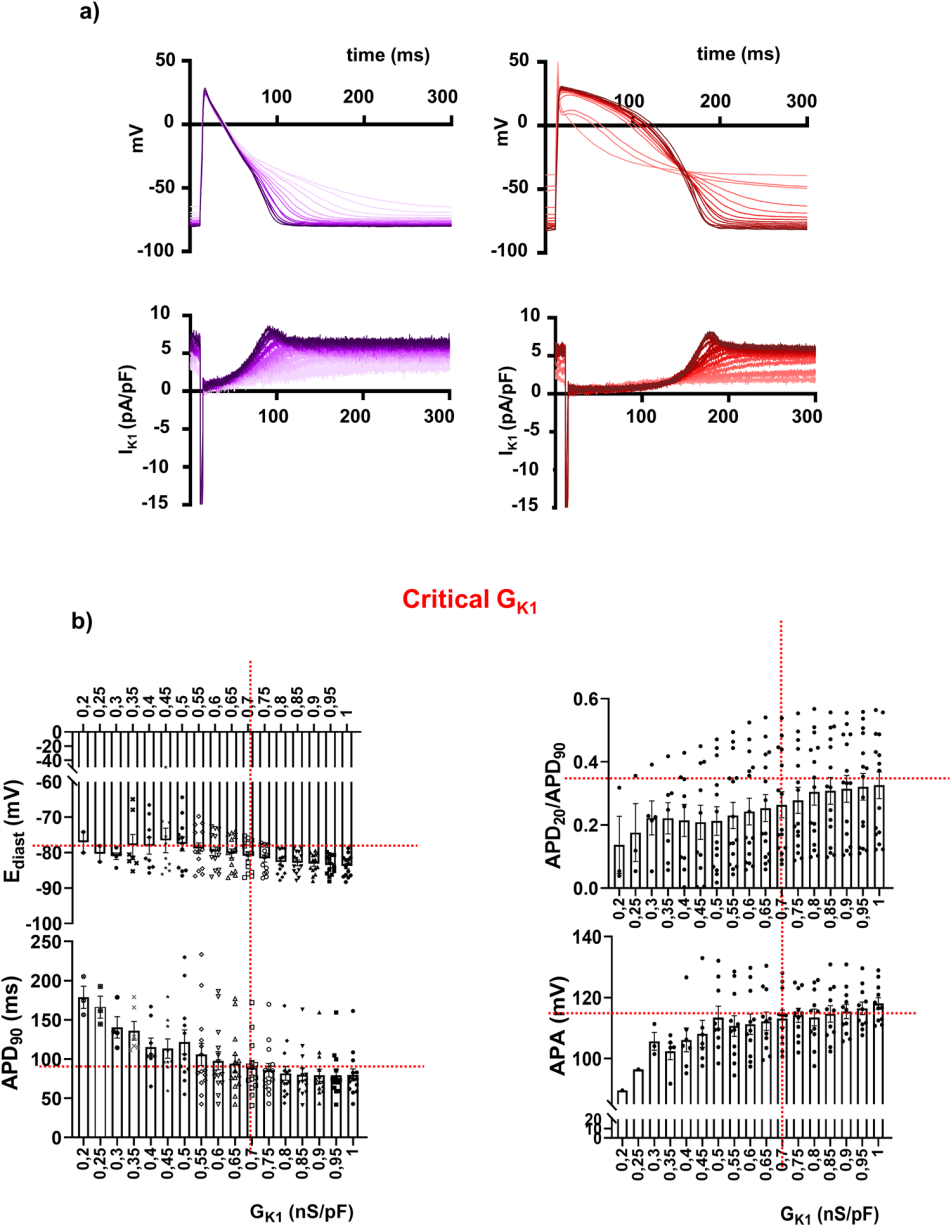
G_K1_ parameter setting of I_K1_Atr_ model. **a** Examples of evoked shorter (atrial-like) and longer (ventricular-like) AP profiles following progressive increase of G_K1_ ranging from 0.2 to 1 nS/pF (0.05 nS/pF step). Light colour code for low G_K1_ values and dark colors code for high G_K1_ values. **b** E_diast_, APD_90_, APD_20_/APD_90_ and APA changes yielded from all cardiomyocytes (*n* = 3 differentiations, *n* = 3-14 cells, depending on the cell stability at lower G_K1_) are represented against G_K1_ values. Red dashed line identifies the critical G_K1_ value (vertical lines) to reach stable AP parameters (horizontal lines shows the steady state of specific parameter obtained injecting critical G_k1_). APD_90_ AP duration measured at 90% of the repolarisation phase, E_diast_ diastolic membrane potential, APD_20_ AP duration measured at 20%, APD_20_/APD_90_ ratio, APA AP amplitude, G_K1_, I_K1_ conductance. Data are presented as mean ± SE.

To obtain a “physiological” AP and overcome the limitation of the low I_K1_ expression in immature hiPSC-CM, we applied two state-of-the-art I_K1_ in silico currents in DC mode: the I_K1_ formulation from the O’Hara-Rudy computational model of human ventricular AP (hereby referred to as I_K1_Ventr_)^16^, and the I_K1_ formulation from the Koivumäki computational model of human atrial AP (hereby referred to as I_K1_Atr_)^18,25^. Both models gave rise to AP that were characterized by different triangulation waveforms hereby referred as “short” an “long” AP durations (Fig. 2a with I_K1_Atr_ and Supplementary Fig. 2a with I_K1_Ventr_). Since the I_K1_Atr_ has never been used in an experimental setting in hiPSC-CM, we sought to define the minimum amount of injectable current (conductance, G_K1_) to obtain a “physiological” AP using this specific model. To this end, we analysed the impact of G_K1_ variation in I_K1_Atr_ taking in account parameters that are considered important electrical markers of AP in mature CM such as E_diast_, APD_90_, APD_20_/APD_90_ ratio and the action potential amplitude (APA). While applying I_K1_Atr_, we progressively increased G_K1_ of 0.05 nS/pF per step (Fig. 2b) and set the value of 0.7 nS/pF as the “critical conductance” to stabilise all four parameters in both short and long AP (Fig. 2a and b, Supplementary Table 2). For completeness, although already known^17^, the critical G_K1_ of 1.9 nS/pF was experimental confirmed in our system for I_K1_Ventr_ (Supplementary Fig. 2 and Table 3).

### O’Hara-Rudy *vs* Koivumäki I_K1_ formulation

**Fig. 3:**
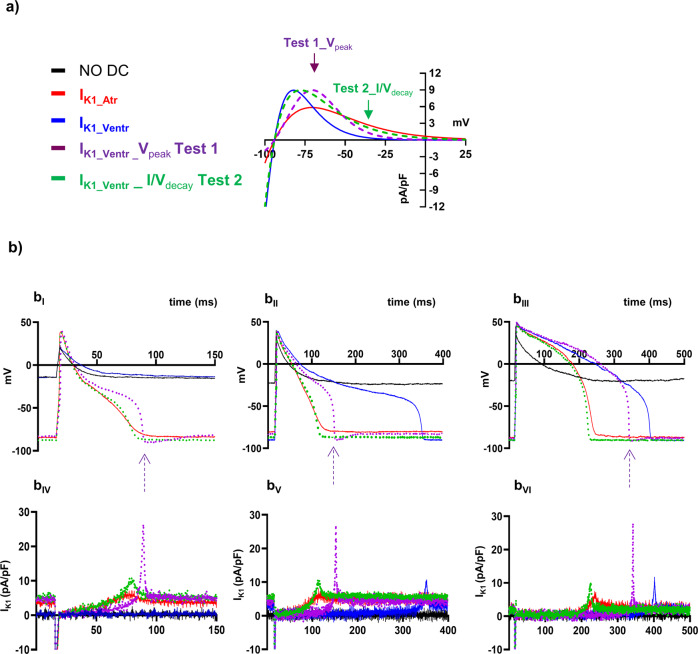
I_K1_Atr_*vs* I_K1_Ventr_ formulation effect on action potential (AP) profile. **a** I_K1_Atr_ and I_K1_Ventr_ computational model I/V relationships; Test-1 and Test-2 representing V_peak_ and I/V_decay_ changes in I_K1_Ventr_ toward those of I_K1_Atr_ (violet and green dotted lines, respectively). **b** AP profiles (b_I-III_) recorded in three hiPSC-CM following the alternative injection of I_K1_Ventr_ (blue), I_K1_Atr_ (red), Test 1_V_peak_ (violet) and Test 2_V_decay_ (green) computational models (b_IV-VI_). I-clamp recordings without I_K1_ injection are represented as black traces in all panels. (*n* = 6–8 cells, *p* < 0.05 see Supplementary Tables 4-5).

To compare the performance of the two formulations in inducing stable AP independently from cell specification, the two in silico I_K1_ currents were alternatively applied in DC mode (real-time switching between models in current clamp) in consecutive recorded cells deriving from both differentiation protocols. I_K1_Atr_ injection resulted in hyperpolarized AP profiles that consistently recapitulate those of adult CM electrical activities in 100% of recorded cells (53/53 cells, 100% red traces in Fig. 3b), characterised by a fast phase-1 repolarisation (Fig. 3b_I_ and 3b_II_) and/or phase-2 plateau (Fig. 3b_III_). Conversely, the injection of I_K1_Ventr_ current, gave rise to prolonged (Fig. 3b_III_) or abnormal plateau phase (Fig. 3b_II_) or often failed to hyperpolarise cells to a physiological E_diast_ (17/53 cells, 32% blue traces in Fig. 3b_I_) similarly to the non-injected CM electrical response (black traces in Fig. 3b_I-_b_III_). To quantitatively assess critical mathematical features that may affect experimental validation of in silico formulation we modified specific parameters allowing approximation of the original I_K1_Ventr_ toward the I_K1_Atr_. First, we changed the voltage dependence of the I/V relation peak bringing it to that of I_K1_Atr_ (from −82 mV to −71 mV, Fig. 3a). Such hybrid model (Test 1_V_peak_, violet dotted line in Fig. 3b_I-III_) still induced abnormal plateau (Fig. 3b_I_ and b_II_) and hyperpolarization towards more negative potentials than diastolic ones, violet arrows, (Fig. 3b_I_-b_III_), in a subset of recorded cells. This effect was ascribable to an excessive I_K1_ injection of current by using Test 1_V_peak_ (Fig. 3b_IV_-b_VI_). Such large peak in this specific test, is due to a larger and steeper phase-3 of AP with respect to the other conditions (Fig. 3b, b_I-III_). Therefore, I_K1_, which is largely inactivated during the AP phase-2, recovers very quickly from inactivation, thus giving rise to the large peaks before that deactivation state. As second test (Test 2_I/V_decay_ green dotted line in Fig. 3), we modified the decay of I_K1_ I/V relationship bringing it to almost complete overlap with that of I_K1_Atr_. The injection of Test-2 model resulted in AP shape that was nearly overlapping that of I_K1_Atr_ as it is confirmed by the statistical analysis performed on all electrical biomarkers (Supplementary Table 4–5), none of those was significantly different between the two models (Test 2 *vs* I_K1_Atr_). Therefore, the I/V decay is crucial to determine the correct AP triangulation in hiPSC-derived CMs. The latter observation was also confirmed by in silico AP-clamp experiments showing that I_K1_Atr_ activates earlier with respect to I_K1_Ventr_ (greater current during the I/V decay), resulting in substantially larger outward current all along the AP duration in cells in which short and/or long AP triangulation was simulated (Supplementary Fig. 3).

### I_K1_Atr_ as instrumental to highlight hiPSC-CM electrical differences

**Fig. 4:**
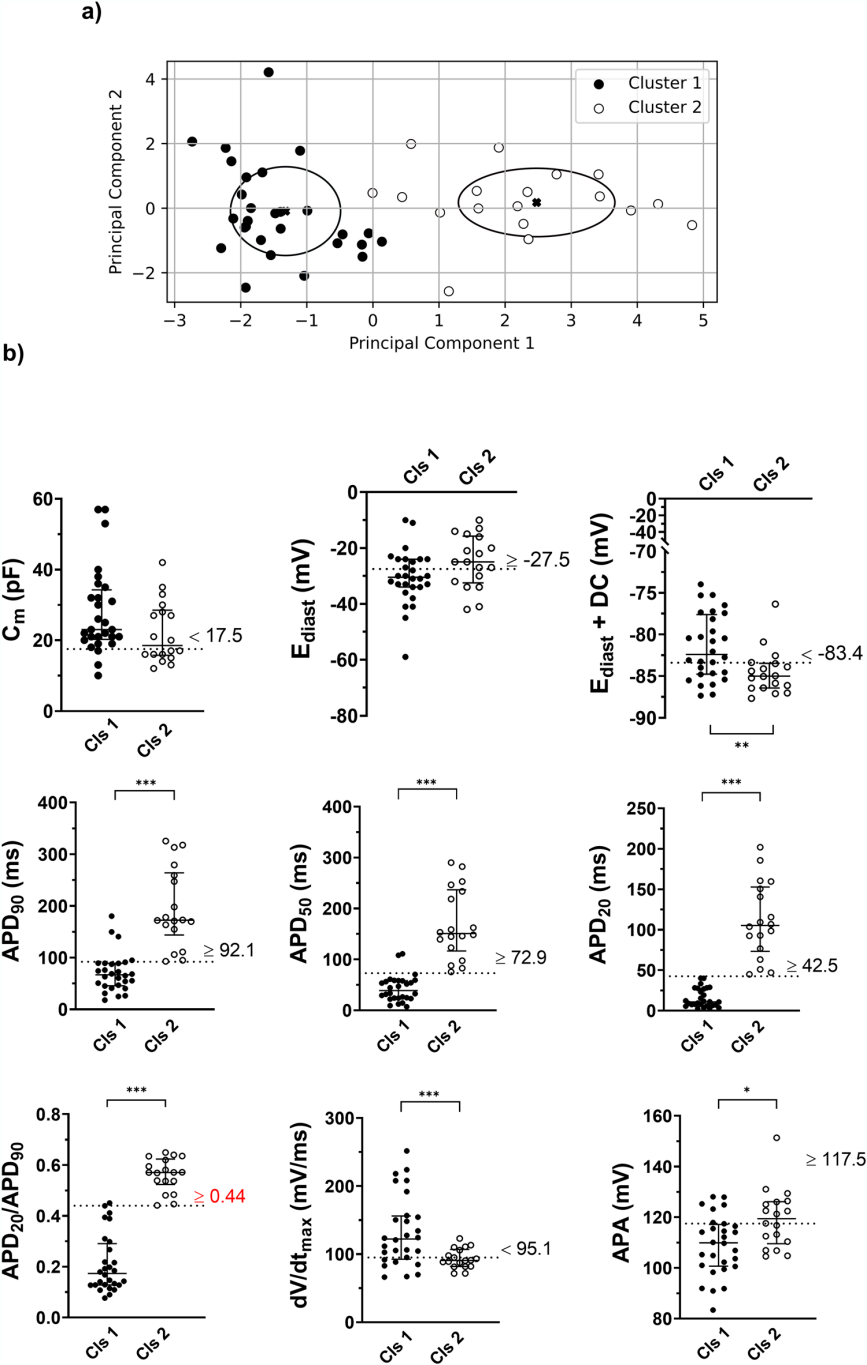
hiPSC-CMs AP phenotype cell classification by unsupervised learning algorithm. **a** A K-means classification algorithm was applied to cluster cells according to 9 electrical parameters (cluster 1 *vs* cluster 2). **b** C_m_, E_diast_, E_diast_ + DC, APD_90_, APD_50_, APD_20_, APD_20_/APD_90_, dV/dt_max_, APA in cluster 1 *vs* cluster 2. The best cut-off discriminating cluster 1 *vs* cluster 2 was defined by ROC curve analysis (see also Supplementary Table 8) and indicated with a dashed line. Data shown are median interquartile range 25^th^, 50^th^, 75^th^ percentiles and analysed by the Mann–Whitney *U* test. C_m_ cell membrane capacitance, E_diast_ diastolic membrane potential, E_diast_ with DC, diastolic membrane potential with dynamic clamp; APD_90_, APD_50_, and APD_20_, action potential duration measured at 90%, 50%, and 20% of the repolarisation phase, respectively, APD_20_/APD_90_ ratio between APD_20_ and APD_90_, dV/dt_max_ maximal AP phase 0 depolarisation velocity, APA AP amplitude; (*n* cells = 46, from 4 independent differentiations). (*p < 0.05; **p < 0.01; ***p ≤ 0.005).

To test the power of I_K1_Atr_ in discriminating the phenotype of differentiated cells, we applied an unsupervised learning algorithm to classify the recorded cells deriving from the two differentiation protocols. Fourty six consecutively recorded cells from 3 independent experiments in which clonally derived cell lines were split in two and differentiated using Std and RA protocols were recorded in DC mode using I_K1_Atr_ formulation with the selected G_K1_ (0.7 nS/pF). Principal component analysis (PCA) was used to reduce the multidimensional parameter space in a two-dimensional plot and visualise cell clustering (Fig. 4a). On the exclusive basis of nine electrical biomarkers cells were divided in two clusters (Cls). We assessed prediction power of each single electrical parameter along the two-dimensional vector (PC1 and PC2, Supplementary Table 6). PC1, which best discriminate clusters, prioritizes APD_90_, APD_20_, APD_50_, and APD_20_/APD_90_, while PC2, which mainly explain variance without an effective discrimination capacity, prioritizes E_diast_, E_diast_ with DC, dV/dt_max_, and APA (Supplementary Table 7). Although the injection of I_K1_Atr_ did not affect the differentiation stage of the two cell-Cls resulted in significant differences among the majority of considered biomarkers. Cls 1 displayed an electrical pattern consistently attributable to the atrial phenotype. Indeed, E_diast_ + DC was less polarised and APD was shorter, with lower amplitude, and more triangular (as shown by lower APD_20_/APD_90_ ratio). On the contrary, cells assigned to Cls 2 were most likely recapitulating a ventricular phenotype showing a significantly higher APD_50_, APD_90,_ APD_20_ as well as an increased APD_20_/APD_90_ ratio (Fig. 4b and Supplementary Table 6; *p* < 0.05). Based on this clustering, we arbitrary defined cells belonging to Cls 1 and Cls 2 as atrial- and ventricular-like cells respectively. We performed a ROC analysis to define a cut-off value that could allow discrimination of cells from Cls 1 *vs* Cls 2 (and hence atrial- *vs* ventricular-like cells) for each electrical biomarker. The best discriminants were APD_20_ (AUC = 1.000 sensitivity and 100% specificity; cut-off 42.5 ms), APD_20_/APD_90_ (AUC = 0.996; 100% sensitivity and 96.4% specificity; cut-off 0.44), APD_50_ (AUC = 0.984; 100% sensitivity and 92.9% specificity; cut-off 72.9 ms), and APD_90_ (AUC = 0.960; 100% sensitivity and 85.7% specificity; cut-off 92.1 ms). Notably, the APD_20_ and, its normalized form, APD_20_/APD_90_ ratio were outperforming all others biomarker in terms of sensitivity and specificity, according to ROC curve analysis (Supplementary Table 8). Moreover APD_20_/APD_90_ ratio displayed the highest OR at univariate analysis (1768 95% CI 1.372–4.299, Supplementary Table 9), thus meaning an increase of 76.8% in likelihood to belong to cluster 2 ventricular phenotype per each unit APD_20_/APD_90_ ratio value (Supplementary Table 10). Thus, we selected APD_20_/APD_90_ ratio (and its cut-off value of 0.44) as the critical biomarker to discriminate atrial- *vs* ventricular-like hiPSC-CM.

### Pharmacological validation of I_K1_Atr_-dependent hiPSC-CM classification

**Fig. 5:**
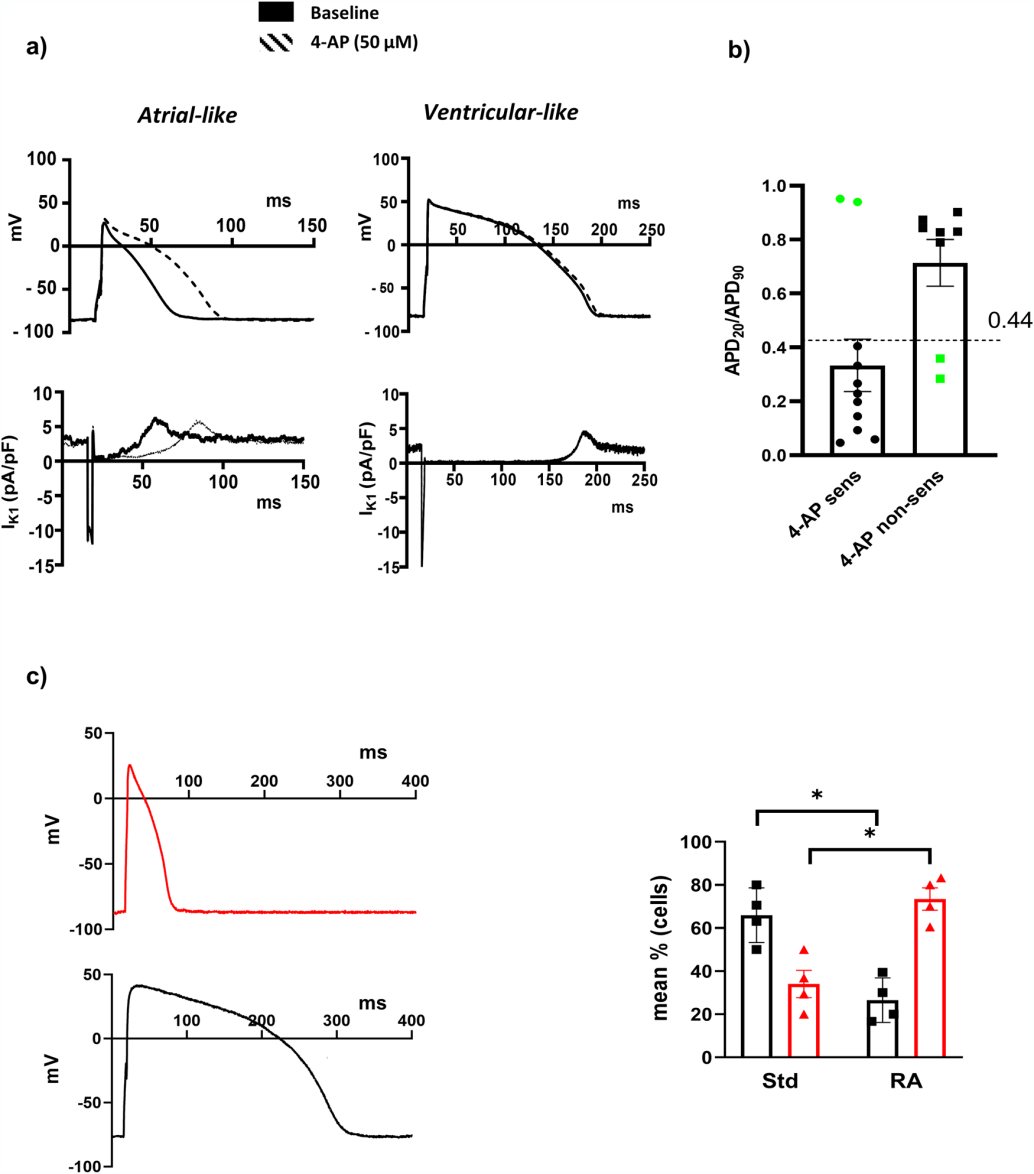
Based-model pharmacological validation and RA efficiency evaluation. **a** Example of cut-off- sensitive and non-sensitive based atrial- and ventricular-like APs recorded during the baseline condition and the superfusion of I_Kur_-specific blocker 4-aminopyridine (4-AP, 50 µM). **b** Distribution of APD_20_/APD_90_ values of 4-AP-sensitive and 4-AP non-sensitive CMs, with respect to the critical cut-off value (0.44). The “failing” cells compared to expectation are showed as green dots in the bar graph (*n* = 19 randomly selected cells from 4 independent differentiations) and AP waveforms are shown in the insets. **c** Atrial- and ventricular-like AP phenotypes represented in the panel and their quantitative change (%) with either the Std- or RA-treated cellular platform (8 independent experiments, 4 Std and 4 RA, each including 20–30 recorded cells). (Std standard protocol, RA retinoic acid, APD_20_/APD_90_ ratio between APD_20_ and APD_90_). Data shown are mean ± SEM (*p < 0.05 paired *t*-test RA *vs* Std).

The APD_20_/APD_90_ cut-off (0.44) defined through unsupervised learning as depicted above was validated pharmacologically in a distinct subset of cells by exploiting the sensitivity of atrial I_Kur_ to a specific dose of 4-AP (50 µM). As expected, 4-AP superfusion caused the prolongation of AP in atrial-like cells (4-AP sensitive cells) whereas it did not affect ventricular-like cells (4-AP non-sensitive cells; Fig. 5a and Supplementary Fig. 4). The percentages of 4-AP sensitive cells were 82% in cells displaying an APD_20_/APD_90_ < 0.44 (thus classified as atrial-like by our model) and 18% in those with an APD_20_/APD_90_ ≥ 0.44 (thus classified as ventricular–like by our model). Overall, the accuracy at experimental validation was about 78.9 % (Fig. 5b). Analysis of 4-AP effects in APD changes showed that the highest prolongation was detected in the APD_20_ phase, where the I_Kur_ mostly contributed during the electrical activity of atrial CM (56.8 ± 20.2%, *n* = 10, Supplementary Table 11).

### Evaluation of differentiation protocol efficiency via I_K1_Atr_

To determine whether the use of the I_K1_Atr_ model is a viable tool for discriminating between atrial and ventricular cells in a mixed population of differentiated CM, we compared the two differentiation protocols (Std and RA) to evaluate their relative efficiency in enriching the two chamber-specific subtypes. A clonally derived iPSC line was splitted in two and subjected to Std and RA differentiation procedures. By applying a previously validated APD_20_/APD_90_ cut-off, 30% of recorded cells in the Std protocol were classified as atrial-like CM. The proportion of atrial–like cells doubled with RA (Fig. 5c).

### I_K1___Ventr_*vs* I_K1___Atr_ application to evaluate pro-arrhythmic features

**Fig. 6:**
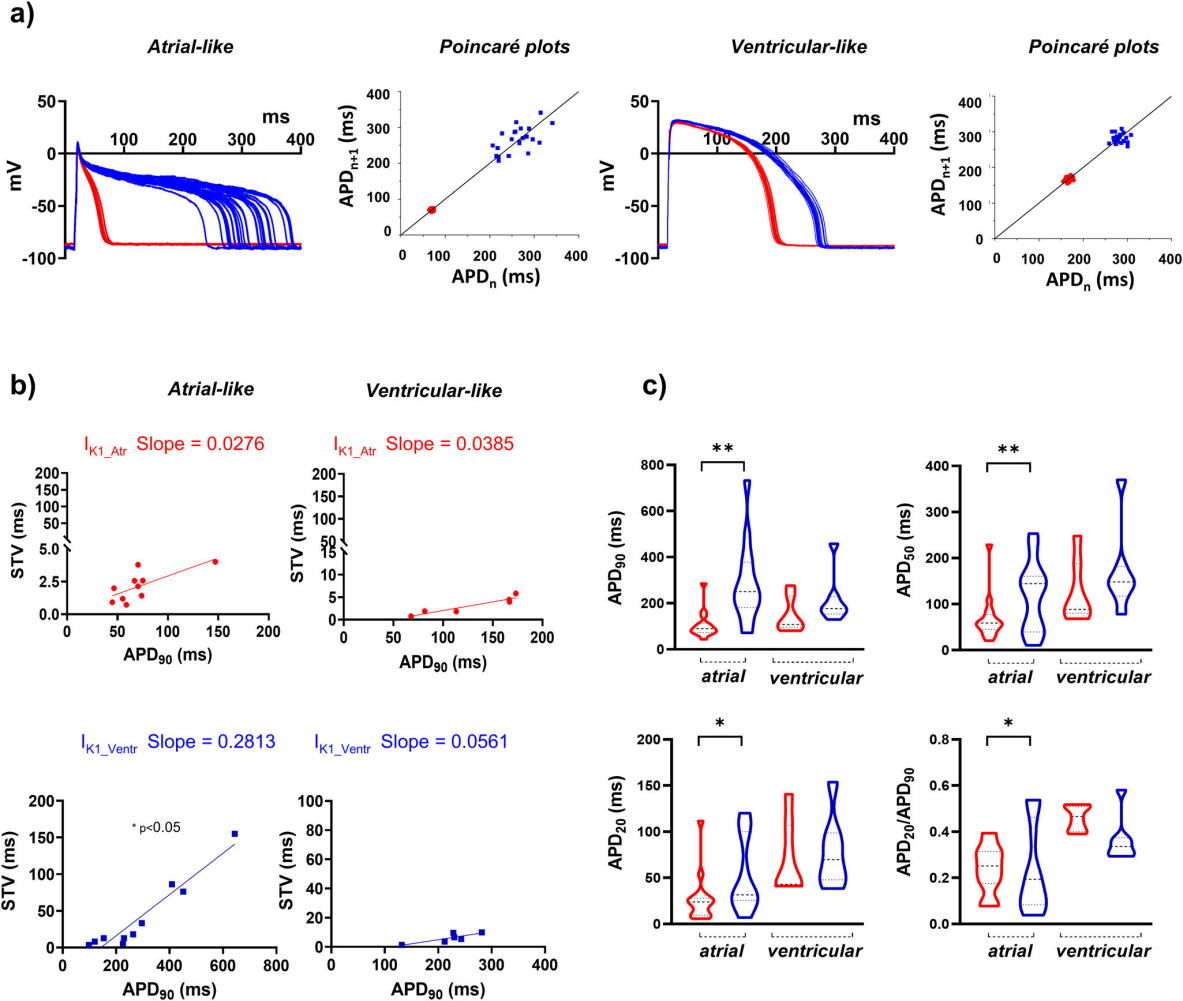
I_K1_Ventr_*vs* I_K1_Atr_ application and repolarization stability. **a** A series of 30 APs recorded under the injection of I_K1_Atr_ or I_K1_Ventr_ computational model either in atrial- or ventricular-like CMs. In parallel, the dispersion of relative APD_90_ values is plotted around the identity line in Poincaré plots. **b** Linear STV/APD_90_ correlations in each condition, resulting significantly higher with I_K1_Ventr_ *vs* I_K1_Atr_ model in atrial- and ventricular-like CMs (*n* = 10 and *n* = 5 cells respectively, from 4 independent differentiations) (*p < 0.05 paired *t*-test RA *vs* Std). **c** Variance comparison of atrial and ventricular (*n* = 16 and *n* = 7 cells respectively, from 2 independent differentiations) electrical biomarkers under the injection of two I_K1_ models, showed as violin graphs. (APD_90_, APD_50_, APD_20_, AP duration measured at 90, 50 and 20% of the repolarisation phase; APD_20_/APD_90_ ratio; I_K1_Ventr_, ventricular I_K1_ equation; I_K1_Atr_, atrial I_K1_ equation; STV, short-term variability). Levene’s test was used to quantify the equality of variance in triangulation parameters (*p < 0.05; **p < 0.01).

To highlight the importance of selecting specific I_K1_ formulation when using DC mode for assessing potential proarrhythmic features of hiPSC-CM, we quantified the beat-to-beat variability of repolarization (BVR), which reflects APD_90_ time-variability (i.e., electrical instability) and represents a pro-arrhythmic parameter^26,27^. BVR was expressed as the short-term variability (STV) of APD_90_, either in atrial- or in ventricular-like CM, under the injection of both models and switching between them in the same cell. An illustrative series of 30 APs recorded in atrial- and ventricular-like CM, during I_K1_Ventr_ (blue trace) and I_K1_Atr_ (red trace) injection, are shown in Fig. 6a–b. Firstly, in the presence of I_K1_Ventr_ injection we observed a high percentage of atrial-like CM that gave rise to an irregular AP plateau (13/23, 56%; blue trace in Fig. 6a), while ventricular-like CM elicited a longer AP plateau (23/30, 76.6%; blue trace in Fig. 6b). The dispersion of APD_90_ values around the identity line in Poincaré plots was increased when I_K1_Ventr_ was injected in atrial cells (blue dots, Fig. 6a). The correlations between STV and APD_90_ were linearly fitted, resulting similar slopes in all conditions except when atrial-like cells were injected with I_K1_Ventr_ model (Fig. 6b). Therefore indicating that in this condition BVR enhancement is not only dependent on APD prolongation^28^. Indeed, these data showed that atrial cells injected with I_K1_Ventr_ elicited an intrinsic condition of rhythm instability. By using I_K1_Ventr_ significant instability was also observed in parameters different from STV (APD_90_, APD_50_, APD_20_ and APD_20_/APD_90_). To quantify such variability, we used the Levene’s test to compare the equality of variance in triangulation parameters while switching between the two I_K1_ models. This analysis further confirmed that the variance of all other parameters significantly deviates from the mean value only when I_K1_Ventr_ is injected in atrial-like cell. Thus, this confirms that the DC mode on atrial-like cells is better suited when running I_K1_Atr_ formulation to inject I_K1_ current (Fig. 6c).

## Discussion

Faced with the difficulty of developing a protocol that can guarantee a pure selection of chamber-specific hiPSC-CMs, it is imperative to develop objective tools that can overcome conventional subjective criteria for phenotype classification. The aim of the current work was to develop an unsupervised learning algorithm that could discriminate the phenotype of differentiated CM based on several electrical biomarkers.

We assessed that the injection of an atrial in silico model of I_K1_ (Koivumäki, I_K1_Atr_) outperformed O’Hara-Rudy’s formulation (I_K1_Ventr_)^25^ in uncovering physiological electrical features of heterogeneous population of hiPSC-CM with mixed distribution of ionic currents, especially when those cells are at depolarised E_diast_. Moreover, in our hands I_K1_Atr,_ had specific advantages in atrial-like cells (e.g. 0% I_K1_Atr_ *vs* 56% I_K1_Ventr_ with irregular AP plateau) whereas in ventricular-like cells both ‘are good’ despite similarly depolarized starting conditions (Fig. 4b). It follows that it also has potential for phenotype discrimination when using DC approach. I_K1_Ventr_ is the best established model of human ventricular AP, and more recent models also inherit its formulation^29–31^. It has been previously applied to study underlying mechanisms of long QT syndrome in hiPSC-CM^17^, however its impact on atrial-like cells has never been properly dissected.

Another important new finding of the present study is that the I/V relationship of I_K1_Ventr_ is characterised by a stronger rectification and higher maximal conductance while, although with lower maximal conductance, the I_K1_Atr_ model has a voltage dependence that evidences a greater outward current density at more depolarised membrane potentials values. To our knowledge, such specific behaviour may reflect a specific propensity of the I_K1_Ventr_ and I_K1_Atr_ models to mimic properties of the endogenous I_K1_ in adult ventricular and atrial CM, respectively^14,15,32^. The injection of I_K1_Atr_ was in all cases associated with a physiological E_diast_ that led to distinct AP profiles whose waveforms’ morphologies ranged from a triangular AP shape with no sustained plateau to a long AP with a spike-and-dome shape, mostly recapitulating either atrial or ventricular human APs^33^. Conversely, I_K1_Ventr_ generated APs characterised by an abnormal “chair-like” plateau when injected in atrial-like cells (Fig. 3). Notably, by using I_K1_Ventr,_ 32% of analysed cells failed to hyperpolarise and trigger a physiological AP. The latter is consistent with the fact that the voltage-dependence of I_K1_Ventr_ only allowed the physiological repolarisation of the most mature cells, characterized by their already more hyperpolarized E_diast_. A new piece of information, that was never considered before, is that the decay of I/V relationship is a crucial determinant in Koivumäk’s formulation. Indeed, by tuning the I/V peak and decay in I_K1_Ventr,_ we empirically verified that while the former induced abnormal plateau phase in atrial-like cells together with an abnormal hyperpolarization (more negative than diastolic value) the I/V decay similar to that of I_K1_Atr_ lead to a more physiological AP triangulation in both atrial- and ventricular-like cells. Thus, at more depolarized potential (from −40 to ~0 mV) it is crucial to have a minor I_K1_ rectification, which results in greater repolarizing I_K1_ amplitude and sudden shortening of the AP. The latter is confirmed by the observation that variance, in triangulation parameters, quantified by Levene’s test, is higher when I_K1_Ventr_ formulation was injected in atrial-like CM. Once optimised, the I_K1_Atr_ model was used as a tool to blindly derive cut-offs of nine biomarkers, which are well-known electrical phenotype indices, to potentially cluster cells within a heterogeneous population of hiPSC-CM. To the best of our knowledge, this is the first time that CM chamber-specificity could be distinguished and classified using DC. A spectral grouping-based algorithm has been used to separate the population of CM dissected from human embryoid bodies into distinct groups based on the similarity of their AP shapes but without the use of DC to make AP more physiological^34^. Bett et al. used DC technology to derive APD_30_ and APD_30_/APD_90_ and by plotting these two parameters they showed that cells fall into two distinct populations, despite the cut-off values to be used as discriminants between cell types were not quantitatively defined^12^.

The unsupervised analysis gave rise to two distinct cell clusters that were significantly different in all values except for the membrane capacitance. Among those biomarkers, the ADP_20_/APD_90_ ratio reached an AUC of 0.996, with a sensitivity and specificity of 100% and 96.4%, respectively. Notably, this parameter has been previously used to distinguish two different AP profiles in human working CM characterised by a typical triangulation for atrial shape and a long plateau for ventricular shape^33^.

This was not surprising as the ADP_20_/APD_90_ ratio is known to discriminate between the fast repolarizing phase of AP (typical of atrial CM) and AP with a long sustained plateau (typical of ventricular CM)^11,35^. About 80% of hiPSC-CM with APD_20_/APD_90_ < 0.44 were sensitive to 4-AP, thus indicating the presence of atrial ionic contributors in these cells. Conversely, cells with APD_20_/APD_90_ > 0.44 responded poorly or did not respond to 4-AP. Two recorded cells had APD_20_/APD_90_ > 0.44 but were still responding to 4-AP. Similarly, 2 out of 10 cells within the 4-AP non-sensitive cells had APD_20_/APD_90_ < 0.44, thus failing the test. The reason for the failing cells in 4-AP experiment can be ascribable to a different grade of maturation of those cells. The ultrarapid delayed rectifier component of K^+^ currents is absent in the early phase of mouse heart embryogenesis and only contribute to repolarization in the postnatal life^36^. Therefore, genes encoding for the channel subunits (i.e KCNA5, encoding the Kv1.5 α-subunit) are finely tuned in the developing heart and very likely the I_Kur_ density progressively increases during the differentiation process of cardiomyocytes. It is plausible that differentiating (but still immature) hiPSC-CM express mixed set of ion channels that affect the repolarization phase of AP in response to specific drug. Moreover, it should be taken into account that the test of 4-AP is measured as the current sensitive to 50 µM^37^, and different concentrations may affect repolarization phase in ventricular CM by acting on different ion channels^38^. As such, the 4-AP at 50 µM may differently affect the repolarization phase of AP in immature CM with reduced I_Kur_ channel density. The latter aspect once again speaks to the point of a population that is heterogeneous along several axes (molecular and electrophysiological) and may partially explain the inconsistency between the expression of specific molecular markers (e.g. myosin light chain isoforms) and the 4-AP test. Starting from the assumption that there are no-clear-cut molecular discriminants to distinguish chamber-specific CMs, the use of AP electrical parameters is desirable to determine the existence of distinct subpopulations of hiPSC-CMs, particularly when chamber-specific phenotype needs to be assigned.

Using this specific cut-off as a critical value to discriminate between atrial- and ventricular-like CM, we confirmed that RA treatment could double the presence of atrial-like hiPSC-CM in culture as compared to Std treatment applied to matched differentiations (73.5 ± 5.1% *vs* 34 ± 6.3%). This is in line with previous work that used immunostaining as a molecular approach to distinguish cell types following fixation^39^.

Our findings were supported by perfusing cells with ACh which activated an ACh-dependent K^+^ current in 88% of RA-treated CM. Treatment led to a prototypical fast cholinergic response that was characterised by a slow decay due to receptor desensitisation, mirroring parasympathetic stimulation in nodal/atrial mature CM^40^. On the contrary, ACh-responsive cells that were a product of the Std differentiation protocol (~47%) showed atypical I_KACh_ kinetics which lacked the fast activation phase, suggesting the presence of a lower affinity ACh-dependent signalling (Fig. 1d). Such pharmacological validation further highlighted the importance of using chamber-specific CM to test drug effects and detect the direct interaction of drugs with specific receptor features^41^.

Finally, by using the BVR as a marker of proarrhythmia^27^ we quantitatively showed that APD_90_ was significantly increased when I_K1_Ventr_ was injected in atrial-like CM under basal conditions, suggesting an intrinsic electrical instability prone to a pro-arrhythmic phenotype. Significant deviation variability was also observed in important electrical AP triangulation parameters. This may lead to a misleading interpretation when iPSC-CM are used as in vitro pro-arrhythmic risk assessment platform and further points out the importance of using an appropriate in silico I_K1_ model when using DC to induce a more mature electrophysiological E_diast_.

A properly hyperpolarised E_diast_ is of paramount importance especially in atrial hiPSC-CM, as these cells differ from human adult atrial CM that possess stronger repolarisation reserve and are less sensitive to changes in the K^+^ rectifying currents^42–44^. Indeed, an “atrial specific” drug (i.e., vernakalant), known to induce prolongation of APD_90_ in atrial hiPSC-CMs^45^, may fail to recapitulate such effects in human atrium^43,45–47^. We believe that by using our approach to assess correct phenotype to undefined hiPSC-CM (most likely matching a specific expression of set of ion channels) and injecting “correct” I_K1_ current may facilitate the activation of specific currents (i.e I_Kur_ that contributes to the repolarization reserve) otherwise functionally hampered by depolarised E_diast._ This could help in the interpretation of specific drug effects.

The use of DC in safety pharmacology is problematic due to the low throughput and complex nature of manual patch clamp in combination with DC. However, several companies focusing on developing automated patch clamp devices to increase throughput and develop new predictive assays using hiPSC-CM (in line with the aims of the Comprehensive in Vitro Proarrhythmia Assay (CiPA) initiative^48^) are devoted to implement their systems with an integrated remote-controlled DC software^49^. We believe that such tool is becoming more and more important in electrophysiology facilities and our experiments shed the light on the fact that the choice of the I_K1_ model to be integrated in such automated software is not trivial and should be taken in serious consideration in order to avoid potential misleading interpretation of results. Our model was validated using one hiPSC line and the most used differentiation protocol towards the atrial lineage; however, given the phenotypic variability among hiPSC-CM from different hiPSC lines, cut-offs and I_k1_ conductance values might require hiPSC-line specific fine-tuning before being universally applicable to multiple hiPSC lines and protocols. Future works will be focussed to empirically determine such critical values.

## Methods

### Generation of hiPSC-CM

#### Cell isolation

iPSCs were obtained by reprogramming adult human dermal fibroblasts (HDFs) of male healthy volunteer. Primary cell lines were derived from a biobank established within previous studies at Cardiocentro Ticino Institute Ente Ospedaliero Cantonale^1,19^. All studies were approved by the local Ethics Committee (Comitato Etico Cantonale, Bellinzona, Switzerland; Ref. CE 2923) and performed according to the Declaration of Helsinki. HDFs were derived as the cellular outgrowth from sternum skin biopsy tissue explants using an ex vivo primary tissue culture technique^50,51^. Briefly, tissue was rinsed with phosphate buffer saline (PBS) and cut into small pieces, treated with TrypLE^TM^/EDTA (SIGMA Life Science) for 2–3 min and finally placed into a 100 mm dish coated with 1% gelatin and cultured in Iscove’s modified Dulbecco’s medium supplemented with 10% foetal bovine serum (FBS) and 1% penicillin-streptomycin (all from Life Technologies). Culture medium was changed twice a week. After 25–30 days of culture, HDFs were enzymatically detached using TrypLE (4 mL) at 37 °C, which was subsequently blocked with culture medium containing 20% FBS. Cells were then centrifuged at 300 g for 5 min, resuspended, and plated onto a gelatin-coated 24 mm dish (Corning; 2.5 × 10^5^ cells per dish). After 48 h, HDFs were reprogrammed into iPSCs.

#### Cellular reprogramming and cardiac differentiation

Two 35 mm dishes of HDFs were used for cellular reprogramming. One dish was used for cell counting and the other was used for infection with Sendai virus carrying OCT3/4, SOX2, KLF4, and MYC (CytoTune™-iPS 2.0 Sendai Reprogramming Kit; Thermo Fisher Scientific), as per manufacturer’s instructions. Medium was changed 24 h later, and then every other day for 1 week. Subsequently, 90% of total cells were transferred into a 60 mm dish coated with Matrigel (hESC Qualified Matrix; Corning), and 10% of total cells were transferred into a second similar dish. Twenty-four hours later, the medium was switched to StemFlex Medium (Miltenyi). The first embryonic stem cell-like colonies appeared 20 to 40 days postinfection, then transferred onto 12-well plates coated with Matrigel (hESC Qualified Matrix; Corning) and expanded.

Cardiac differentiation was induced using Gibco™ PSC Cardiomyocyte Differentiation Kit (Miltenyi). hiPSC colonies at passage P8-P10 were induced to differentiate into cardiomyocytes in feeder-free conditions using a “standard” protocol (Std) via modulation of canonical Wnt signalling or applying retinoic acid (RA) to induce atrial differentiation^20,23^. The latter included the addition of 1 µM RA at stages d4–d8 of the differentiation protocol. Both protocols included a cardiac-specific metabolic selection step by replacing glucose with lactate from d11 to d13 (Supplementary Fig. 1a)^52^. Spontaneously beating cells were maintained in RPMI (Gibco) supplemented with B27 (Gibco) at 37 °C. Medium was changed every second day. Two clonally derived hiPSC lines from the same subject were used as cell source to obtain hiPSC-CMs using the two differentiation protocols (Std and RA). For electrophysiology experiments “n” refers to number of recorded single cell/cluster, deriving from independent differentiations. Independent experiments (differentiations) are indicated in the method and in the figure legends for each experiment.

### Molecular characterisation

#### Immunofluorescence

Cells were fixed with 4% paraformaldehyde and stained with primary antibody overnight (MLC2v; Proteintec 10906-1-AP and MLC2a; Synaptic System 311011). Alexa Fluor secondary antibody (Thermo Fisher Scientific) was used for detection. Immunostained cell culture images (*n* = 4 independent differentiations) were acquired with a Lionheart FX automatic microscopy at 10× magnification and analysed with Gen5 software (Biotek).

#### RNA extraction, reverse transcription, and real-time PCR

hiPSC-CMs were lysed with TRI Reagent (Sigma) as per manufacturer’s instructions. The pellet was air-dried, resuspended in DEPC water, and RNA was quantified with a NanoDrop™ 2000 c (Thermo Fisher Scientific). RNA (500 ng) was reverse-transcribed using GoScript™ Reverse Transcription System (Promega). Real-time analysis was performed on a CFX connect Real-time PCR detection system (Bio-Rad). Data are shown as 2^−ΔΔCt^ values (*n* = 9 technical replicates from 4 independent differentiations). Coupled primers were as follows: *COUP-TFI forward: AAGCCATCGTGCTGTTCAC, Reverse: GCTCCTCAGGTACTCCTCCA; COUP-TFII forward: CCGAGTACAGCTGCCTCAA, Reverse: TTTTCCTGCAAGCTTTCCAC; KCNA5 forward: CGAGGATGAGGGCTTCATTA, Reverse: CTGAACTCAGGCAGGGTCTC; KCNJ3 forward: AAAAACGATGACCCCAAAGA, Reverse: TGTCGTCATCCTAGAAGGCA; GAPDH forward: TGCACCACCAACTGCTTAGC, Reverse: GGCATGGACTGTGGTCATGAG*.

### Electrophysiology and contractility

#### Multielectrode array (MEA) recordings

Extracellular field potentials (FPs) were recorded from microdissected, spontaneously beating cell clusters, at d30 of differentiation. MEA dishes were coated with 10 µg/ml Synthemax (Corning) and 0.02% gelatin and incubated for 2 h at 37 °C. Clumps (200–300 µm) of beating cells were microdissected using surgical scissors and positioned on the electrodes of standard 60 electrode MEAs at high spatial (200 µm) resolution (60MEA-200/30iR-Ti, Multi Channel Systems, Reutlingen, Germany). FPs were recorded after at least 72 h after plating to allow for cell attachment. During recordings, the temperature was maintained at 37 °C. FP duration was analysed offline by Clampfit (Molecular Devices), as reported^52^. Corrected FP duration (FPDc) was calculated using the standard Bazett’s formula (*n* = 10 and *n* = 17 clusters of Std and RA respectively, from 6 independent differentiations).

#### Contractility measurements

Contractility was assessed in spontaneously beating cell clusters seeded on MEAs as previously described^52,53^. Briefly, beating clusters of both Std- and RA-hiPSC-CM (*n* = 7 clusters of Std and RA, from 6 independent differentiations) were microdissected and seeded on standard 60 electrode MEAs (Multi Channel Systems). Movies of beating clusters were acquired with a Thorlabs DCC3240M CMOS camera at 120 fps together with MEA FP recordings. The movies were converted to raw AVI using *ffmpeg* (www.ffmpeg.org) and the contractile properties were quantified using the MUSCLEMOTION ImageJ macro^53^. FP and contraction traces were plotted as synchronised.

#### V-clamp measurements for ACh sensitivity

Acetylcholine-activated inward-rectifying potassium current (I_KACh_) was elicited by ACh 10 uM in V-clamped cells at −40 mV in the presence of 5 uM L type Ca^2+^ channel blocker nifedipine.

#### APs measurements

Dissociated hiPSC-CMs at ≥d30 of differentiation were plated onto 35 mm dishes at very low density and electrophysiologically analysed after 3 days. APs were acquired with MultiClamp 700 B amplifier (Molecular Devices) connected to Digidata 1550 A (Molecular Devices) and filtered at 1 kHz via pClamp 10.6 (Molecular Devices). APs were stimulated at 1 Hz during superfusion of Tyrode’s solution: 154 mM NaCl, 4 mM KCl, 2 mM CaCl_2_, 1 mM MgCl_2_, 5.5 mM D-glucose, and 5 mM HEPES-NaOH (pH 7.35). Experiments were carried out in whole cell configuration (seal resistance: 2.3 ± 0.09 GΩ; access resistance: 18.8 ± 0.8 MΩ) at 35 °C; the pipette solution contained: 23 mM KCl, 110 mM KAsp, 0.4 mM CaCl_2_, 3 mM MgCl_2_, 5 mM HEPES-KOH, 1 mM EGTA-KOH, mM 0.4 NaGTP, 5 mM Na_2_ATP, and 5 mM Na_2_PC. There was no current injection to compensate for leakage. Nine biomarkers were measured from AP recordings: cell membrane capacitance (C_m_), E_diast_, E_diast_ with DC, AP duration measured at 90%, 50%, and 20% of the repolarisation phase (APD_90_, APD_50_, and APD_20_, respectively), APD_20_/APD_90_ ratio, maximal AP phase 0 depolarisation velocity (dV/dt_max_), and AP amplitude (APA). Beat-to-beat variability of repolarisation duration was expressed as the short-term variability (STV) of APD_90_ (i.e., the mean orthogonal deviation from the identity line in the APD_n_ *vs* APD_n+1_ Poincaré plot)^27,54^, and calculated as follows: STV = ∑〖(|〖APD〗_(*n* + 1)-〖APD〗_n | /[n_(beats) x √2] 〗for 30 consecutive APs (nbeats) at steady-state.

#### DC recordings

APs, recorded from the hiPSC-CMs, were acquired at a sampling rate of 5 kHz to drive the numerical I_K1_ model in DC. Modelled I_K1_ was calculated in real-time (within one sampling interval) and injected into the myocyte during continued AP recording. To implement DC, the Multiclamp 700 B amplifier (Axon Instruments) was connected to a data acquisition board (DAQ, 6024E PCI, National Instruments) on a personal computer (Intel Celeron 3.20 GHz). The open-source Real-Time Experiment Interface (RTXI) was used: it is a fast and versatile real-time biological experimentation system based on Real-Time Linux. System features and custom user code were implemented as modules written in C + + ^55^.

#### In silico I_K1_

We considered two I_K1_ formulations from two of the most recent human AP models. For the ventricular I_K1_ the equation was taken from the ORd model (Eq. 5; I_K1_Ventr_)^16^, while for the atrial I_K1_ the equation was from Koivumäki et al. (Eq. 7; I_K1_Atr_)^25^, which has inherited the expression from the parent model of Nygren et al.^18^:

1 $${x}_{K1,\infty }=\frac{1}{1+{e}^{\left(-\frac{V+2.5538\cdot {[{K}^{+}]}_{o}+144.59}{1.5692\cdot {[{K}^{+}]}_{o}+3.8115}\right)}}$$

2 $${\tau }_{x,K1}=\frac{122.2}{{e}^{\left(\frac{-(V+127.2)}{20.36}\right)}+{e}^{\left(\frac{V+\left.236.8\right)}{69.33}\right)}}$$

3 $$\frac{d{x}_{K1}}{dt}=\frac{{x}_{K1,\infty }-{x}_{K1}}{{\tau }_{x,K1}}$$

4 $$\,{R}_{K1}=\frac{1}{1+{e}^{\left(\frac{V+105.8-2.6\cdot {[{K}^{+}]}_{o}}{9.493}\right)}}\\ {\underline{{G}_{K1}}}=1.908 * scalin{g}_{DC}\,\frac{nS}{p F}$$

5 $${I}_{K1\_Ventr}={\underline{{G}_{K1}}}\cdot \sqrt{{[{K}^{+}]}_{o}}\cdot {x}_{K1}\cdot {R}_{K1}\cdot (V-{E}_{K})$$

6 $${\underline{{G}_{K1}}}=0.7 * scalin{g}_{DC}\,\frac{nS}{pF}$$

7 $$\,{I}_{K1\_Atr}=\,\frac{{\underline{{G}_{K1}}}\cdot {[{K}^{+}]}_{o}^{0.4457}\cdot (V-{E}_{K})\,}{\left(1+{e}^{\left(1.5\cdot (V-{E}_{K}+3.6) * \frac{\frac{F}{R}}{T}\right)}\right)}$$

where V and ${E}_{K}$ denote the membrane potential and K^+^ equilibrium potential (in mV) respectively; ${E}_{K}$ was set to –94.7 mV based on K^+^ concentration in external bath $({[{K}^{+}]}_{o})$ and pipette solutions (see *APs measurements*); $\underline{{G}_{K1}}$ is the maximal conductance, which has been varied using the dimensionless scaling factor, $scalin{g}_{DC}$: from 0.2 to 1 for I_K1_Atr_ (with a 0.05 step) and from 0.4 to 2 for I_K1_Ventr_ (with a 0.1 step), respectively (*n* = 3-14 cells for I_K1_Atr_ and *n* = 3-9 cells for I_K1_Ventr_, depending on the cell stability at lower GK1, from 3 independent differentiations). The DC implementation, based on “modules” architecture, allows the user to switch in real-time the I_K1_ formulation to be injected into the cell and to analyse its response in terms of AP waveform changes. To assess which is the feature that affect the most the different experimental results obtained with I_K1_Ventr_ and I_K1_Atr_, we also defined two additional synthetic I_K1_ formulations called Test 1_V_peak,_ and Test 2_I/V_decay_ (see ‘*O’Hara-Rudy vs Koivumäki I*_*K1*_ *formulation*’ Results subsection and Fig. 3a), by changing the following parameters:

8 $$\,{x}_{K1,{\infty }_{Tes{t}_{1}}}=\frac{1}{1+{e}^{\left(-\frac{V+2.5538\cdot {[{K}^{+}]}_{o}+57.836}{1.5692\cdot {[{K}^{+}]}_{o}+3.8115}\right)}}$$

9 $$\,{\underline{{G}_{K{1}_{Tes{t}_{1}}}}}=6.417\frac{nS}{pF},$$

and

11 $$\,{R}_{K{1}_{Tes{t}_{2}}}=\frac{1}{1+{e}^{\left(\frac{V+135.8-2.6\cdot {[{K}^{+}]}_{o}}{15.493}\right)}}$$

12 $$\,\underline{\,{G}_{K{1}_{Tes{t}_{2}}}}=\,6.258\,\frac{nS}{pF},$$

respectively, in the ORd model.

The DC implementation, based on “modules” architecture, allows the user to switch in real-time the I_K1_ formulation to be injected into the cell and to analyse its response in terms of AP waveform changes.

### Cell clustering and unsupervised algorithm

For the unsupervised analysis electrical parameters recorded from 46 consecutively recorded cells deriving from 4 independent experiments were used as input. The analysis was carried out through the following processing chain: Principal component analysis (PCA) was used to reduce the number of I-Clamp recorded parameters from nine to two (two-dimensional plot) and visualise cells clustering according to an electrical pattern. Then, a k-means algorithm has been applied to cluster the cells into two groups; the inputs of this algorithm are the cells projected in the new space produced by the PCA. This algorithm created “2” groups clusters according to electrical parameters similarities. Specifically, k-means minimized the intra-cluster distance between each cell belonging to the same group.

### Pharmacological validation

The discriminating power of APD_20_/APD_90_ ratio (cut-off, 0.44) was validated pharmacologically in a distinct subset of cells (*n* = 19 randomly selected cells from 4 independent differentiations) by exploiting the sensitivity of atrial I_Kur_ to a specific dose of 4-AP (50 µM). AP, 4-AP (Sigma-Aldrich) was added to the Tyrode’s solution at 50 µM and biomarkers recorded see above^56^. To determine whether the use of the I_K1_Atr_ model is a viable tool for discriminating between atrial and ventricular cells in a mixed population of differentiated CM, we compared the two differentiation protocols to evaluate their relative efficiency in enriching the two subtypes.

Electrical biomarkers (see above) were yielded from 20–30 cells from and 8 independent experiments including 4 Std and 4 RA differentiation (see legend Fig. 4).

### Statistics and reproducibility’

Data are expressed as the mean ± standard error of mean (SEM). The differences between groups were tested with paired *t*-test analysis as appropriate. Post-hoc comparison between individual means was performed with the Bonferroni test. A *p*-value <0.05 was considered statistically significant. For the correlation between STV and APD_90_, a linear regression analysis that showed a 95% confidence band of the best fit trend line was performed. Levene’s test was used to quantify the equality of variance in triangulation parameters.

AP parameters distribution was assessed by the Kolmogorov-Smirnov test. Non-normally distributed data was expressed as median and interquartile range (25^th^, 50^th^, and 75^th^ percentiles) and analysed by the Mann–Whitney *U* test. Diagnostic performance of single electrical biomarkers was evaluated by analysis of receiver operating characteristic (ROC) curves; the area under the curve was reported together with the 95% confidence interval. The cut-off correspondent to the maximum accuracy was derived by the Youden Index (J = Sensitivity + Specificity-1).

### Reporting summary

Further information on research design is available in the Nature Portfolio Reporting Summary linked to this article.

## Supplementary information

Supplementary Information

Reporting Summary

## Data Availability

Datasets generated during and/or analysed during the current study are available in the github.com repository (https://github.com/CardiovascularTheranostics/DynamicClamp.git). All statistical analyses generated during this study are included in this published article (and its supplementary information files).
