# Supplementary material for: A dynamic clamping approach using in silico IK1 current for discrimination of chamber-specific hiPSC-derived cardiomyocytes
Source: Commun Biol. 2023 Mar 18;6:291. doi: 10.1038/s42003-023-04674-9 (PMC10024709; doi:10.1038/s42003-023-04674-9)
Supplement: Supplementary file 1 — Supplementary Information [file 42003_2023_4674_MOESM1_ESM.pdf]

A dynamic clamping approach using *in silico* I_{K1} current for discrimination of chamber-specific hiPSC-derived cardiomyocytes.

Claudia Altomare[1,2]; Chiara Bartolucci[3]; Luca Sala[4]; Carolina Balbi[2,5,6]; Jacopo Burrello[1,7]; Nicole Pietrogiovanna[1]; Alessio Burrello[8]; Sara Bolis[1,2,5]; Stefano Panella[1,2]; Martina Arici[9]; Rolf Krause[10]; Marcella Rocchetti[9]; Stefano Severi*[3]; Lucio Barile*[1,2,10,11]

[1]Cardiovascular Theranostics, Istituto Cardiocentro Ticino, Ente Ospedaliero Cantonale, Lugano, Switzerland. [2]Laboratories for Translational Research, Ente Ospedaliero Cantonale, Bellinzona, Switzerland. [3]Department of Electrical, Electronic and Information Engineering 'Guglielmo Marconi', University of Bologna, Cesena, Italy. [4]Istituto Auxologico Italiano IRCCS, Center for Cardiac Arrhythmias of Genetic Origin and Laboratory of Cardiovascular Genetics, Milan, Italy. [5] Cellular and Molecular Cardiology, Istituto Cardiocentro Ticino, Ente Ospedaliero Cantonale, Lugano, Switzerland. [6]Center for Molecular Cardiology, University of Zurich, Zurich Switzerland. [7]Division of Internal Medicine 4 and Hypertension Unit, Department of Medical Sciences, University of Turin, Italy. [8]Department of Electrical, Electronic and Information Engineering (DEI), University of Bologna, Italy. [9]Department of Biotechnology and Biosciences, Università degli Studi di Milano-Bicocca, Milano, Italy. [10]Faculty of Informatics, Università Svizzera Italiana, Lugano, Switzerland.[11]Faculty of Biomedical Sciences, Università Svizzera Italiana, Lugano, Switzerland.[12]Institute of Life Science, Scuola Superiore Sant'Anna, Pisa, Italy.

*Corresponding Authors

Addresses for correspondence:

Lucio Barile, PhD
Cardiocentro Ticino Institute
Ente Ospedaliero Cantonale
Via Tesserete 48
6900 Lugano, Switzerland
+41 918053384
lucio.barile@eoc.ch

Stefano Severi, PhD
University of Bologna
Via Guglielmo Marconi, 10,
40122 Bologna BO, Italy
stefano.severi@unibo.it

Table 1

Parameters	Std	RA	# clusters	
			Std	RA
**Rate (Hz)**	0.59 ± 0.12	1.3 ± 0.16 *	7	10
**Contraction duration (ms)**	819.8 ± 127.6	343.9 ± 32.66 **	7	7
**Time to peak (ms)**	344.2 ± 58.3	130.3 ± 30.7 **	6	6
**Relaxation time (ms)**	482.1 ± 94.6	199.4 ± 21.2 *	7	7

Supplementary Table 1. Contraction parameters from MM of spontaneous beating hiPSC-CM from Std and RA differentiation protocol. Number of clusters have been analysed in different differentiation wells from 4 experiments. (Data are presented as mean±SE)

Table 2

G_{K1} (nS/μF)	E_{diast} (mV)	APD_{90} (ms)	APD_{20}/ APD_{90}	APA (mV)	# cells
**1**	-83.4 ± 0.7	78.5 ± 8.2	0.326 ± 0.04	118.1 ± 1.9	13
**0.95**	-83.5 ± 0.7	79.4 ± 7.5	0.321 ± 0.04	116.5 ± 2.1	14
**0.9**	-83.1 ± 0.7	79.1 ± 7.6	0.314 ± 0.04	115.4 ± 2.3	14
**0.85**	-82.8 ± 0.7	80.3 ± 8.2	0.308 ± 0.04	114.7 ± 2.7	14
**0.8**	-82.5 ± 0.8	81.4 ± 8.7	0.305 ± 0.04	113.5 ± 2.7	14
**0.75**	-81.6 ± 0.9	85.5 ± 8.6	0.27 ± 0.04	114.2 ± 2.3	14
**0.7**	-80.8 ± 1.1	88.6 ± 9.2	0.26 ± 0.04	113.1 ± 2.8	14
**0.65**	-80.1 ± 1.2	93.7 ± 10.5	0.25 ± 0.04	112.2 ± 3	14
**0.6**	-79.6 ± 1.3	97.3 ± 11.4	0.24 ± 0.04	111.3 ± 3.3	14
**0.55**	-78.7 ± 1.4	105.6 ± 14.1	0.23 ± 0.04	110-7 ± 3.3	14
**0.5**	-77.4 ± 1.9	121.3 ± 15.4	0.212 ± 0.04	113.4 ± 3.7	12
**0.45**	-75.8 ± 3.4	112.7 ± 12	0.208 ± 0.05	108.1 ± 4.4	9
**0.4**	-77.1 ± 2.4	114.6 ± 11.1	0.214 ± 0.05	106.1 ± 3.9	8
**0.35**	-76.7 ± 3.2	134.4 ± 11.1	0.221 ± 0.05	102.4 ± 2.7	6
**0.3**	-78.9 ± 2.8	137.2 ± 10.7	0.222 ± 0.05	105.6 ± 2.9	4
**0.25**	-76.2 ± 5.7	161.7 ± 9.8	0.17 ± 0.09	95.4	1-3
**0.2**	-73 ± 6	174.6 ± 10.3	0.137 ± 0.09	89.5	1-3

Supplementary Table 2. I_{K1_Atr} G_{K1} optimization for E_{diast}, APD_{90}, APD_{20}/APD_{90} and APA stabilization. Progressive values of four parameters yielded under the increase of the conductance (G_{K1}) of 0.05 nS/μF per step. (G_{K1}, I_{K1} maximal conductance; E_{diast}, diastolic membrane potential; APD_{90}, action potential duration at 90% of repolarization; APD_{20}/APD_{90} ratio; APA, Action Potential Amplitude).

Table 3

G_{K1} (nS/µF)	E_{diast} (mV)	APD_{90} (ms)	# cells
**2**	-89.88 ± 0.2	87.7 ± 30	9
**1.9**	-89.68 ± 0.7	92.05 ± 30.7	9
**1.8**	-89.64 ± 0.2	98.8 ± 33.8	9
**1.7**	-89.61 ± 0.7	99.36 ± 30.9	9
**1.6**	-89.38 ± 0.3	105.6 ± 31.8	9
**1.5**	-89.42 ± 0.3	112.7 ± 32	9
**1.4**	-89.6 ± 0.26	126.3 ± 34.4	7
**1.3**	-89.16 ± 0.39	139.8 ± 38.3	8
**1.2**	-89.06 ± 0.38	184.8 ± 59.6	8
**1.1**	-88.95 ± 0.52	139.4 ± 43.2	7
**1**	-88.73 ± 0.46	148.2 ± 46.1	7
**0.9**	-88.53 ± 0.47	159.1 ± 32	7
**0.8**	-88.24 ± 0.52	172.5 ± 47	7
**0.7**	-88.14 ± 0.6	112.7 ± 32	4-6
**0.6**	-87.61 ± 0.74	311 ± 68.2	4-6
**0.5**	-86.41 ± 1.26	314.16 ± 91.4	3-4
**0.4**	-86.32 ± 1.4	387.7 ± 75.5	3-4

Supplementary Table 3. I_{K1_Ventr} G_{K1} optimization for E_{diast} and APD_{90} stabilization. Progressive values of E_{diast} and APD_{90} under the increase of the conductance (G_{K1}) of 0.1 nS/µF per step. (G_{K1}, I_{K1} maximal conductance; E_{diast}, diastolic membrane potential; $APD_{90,}$ action potential duration at 90% of repolarization).

Table 4

**Atrial-like CMs**	**K** **(8 cells)**	**Ord** **(6 cells)**	**Test 1** **(7 cells)**	**Test 2** **(7 cells)**
E_{diast}	-84.3 ± 1.6	-89.1 ± 1.3	-85.7 ± 0.8	-88.2 ± 0.7
APD_{90} (ms)	82.1 ± 8.2	346.6 ± 90	123.9 ± 19.2	77.6 ± 8.6
APD_{50} (ms)	49.2 ± 6.4	164.3 ± 30.7	78.2 ± 15	55.2 ± 8.1
APD_{20} (ms)	15.7 ± 2.8	23.5 ± 4.2	18.1 ± 4.3	17.5 ± 3.5
APD_{20}/APD_{90}	0.19 ± 0.03	0.1 ± 0.01	0.17 ± 0.03	0.3 ± 0.02
dV/dt_{max} (mV/ms)	161.5 ± 19	185.1 ± 32.2	164.7 ± 16.1	183 ± 24.5
APA (mV)	122.6 ± 2.1	128.3 ± 2.2	128.2 ± 3.4	126 ± 1.2
**Ventricular-like CMs**	**K** **(5 cells)**	**Ord** **(5 cells)**	**Test 1** **(5 cells)**	**Test 2** **(5 cells)**
E_{diast}	-88 ± 14.7	-90.6 ± 0.1	-88 ± 0.2	-90.2 ± 0.3
APD_{90} (ms)	146.6 ± 36.8	242.5 ± 60.6	172.7 ± 41	130.4 ± 28.6
APD_{50} (ms)	126.2 ± 3.2	192.6 ± 45.6	155.5 ± 37.8	116.2 ± 27.1
APD_{20} (ms)	68.2 ± 19.3	81.9 ± 19.2	66.6 ± 19	63.6 ± 17.5
APD_{20}/APD_{90}	0.45 ± 0.03	0.33 ± 0.01	0.4 ± 0.02	0.5 ± 0.02
dV/dt_{max} (mV/ms)	168.8 ± 34.2	181.2 ± 27	175.3 ± 25	164.2 ± 26.1
APA (mV)	126.8 ± 3.2	129.4 ± 23	130 ± 2.9	127.4 ± 2.6

Supplementary Table 4. O'Hara Rudy *vs* Koivumäki tests: electrical biomarker analysis. E_{diast} with DC, diastolic membrane potential with Dynamic Clamp; $APD_{90,}$ APD_{50} and APD_{20}; action potential duration at 90, 50 and 20% of repolarization; ADP_{20}/APD_{90} ratio; dV/dt_{max}, maximum AP upstroke velocity; APA, AP amplitude.

Table 5

Atrial-like CMs	K *vs* T1	K *vs* T2	ORd *vs* T1	ORd *vs* T2
APD_{90} (ms)	p = 0.044*	p = 0.7	p = 0.04*	p = 0.008**
APD_{50} (ms)	p = 0.026 *	p = 0.56	p = 0.04*	p = 0.003**
APD_{20} (ms)	p = 0.39	p = 0.69	p = 0.6	p = 0.29
APD_{20}/APD_{90}	p = 0.64	p = 0.5	p = 0.02*	p = 0.00003**
dV/dt_{max} (mV/ms)	p = 0.92	p = 0.13	p = 0.58	p = 0.9
APA (mV)	p = 0.17	p = 0.25	p = 0.9	p = 0.4
**Ventricular-like CMs**	**K *vs* T1**	**K *vs* T2**	**ORd *vs* T1**	**ORd *vs* T2**
APD_{90} (ms)	p = 0.63	p = 0.7	p = 0.33	p = 0.1
APD_{50} (ms)	p = 0.56	p = 0.81	p = 0.54	p = 0.18
APD_{20} (ms)	p = 0.95	p = 0.86	p = 0.58	p = 0.5
APD_{20}/APD_{90}	p = 0.85	p = 0.57	p = 0.2	P = 0.017*
dV/dt_{max} (mV/ms)	p = 0.06	p = 0.9	p = 0.9	p = 0.66
APA (mV)	p = 0.49	p = 0.87	p = 0.91	p = 0.61

Supplementary Table 5. Statistical evaluation of the impact of two distinct features of I_{K1_Atr} *vs* I_{K1_Ventr} on electrical biomarkes. Starting from the original I_{K1_Ventr}, we modified critical parameters to approximate its formulation toward the I_{K1_Atr}, thus highlighting critical features of such model. In particular, Test-1 and Test-2 represent V_{peak} and V_{decay} changes in I_{K1_Ventr} toward those of I_{K1_Atr} (see Figure 3a). The four different I_{K1} formulations were consecutively injected in a subset of cells. The statistical significance of each comparison between the biomarkers upon injection of different I_{K1} formulations is reported in the Table for atrial- and ventricular-like CMs (see Table 4).

Table 6

**Parameters**	**Cluster 1** [n=28]			**Cluster 2** [n=18]			***P*-value**
	**25th**	**50th**	**75th**	**25th**	**50th**	**75th**	
C_m (pF)	20	23	34	16	19	29	0.061
E_{diast} (mV)	-34	-30	-24	-33	-25	-16	0.169
E_{diast} with DC	-84.77	-82.41	-77.62	-86.44	-85.01	-83.47	0.008**
APD_{90} (ms)	45.61	66.66	89.07	143.88	172.47	264.07	<0.001***
APD_{20} (ms)	6.76	10.91	27.52	73.39	105.25	152.83	<0.001***
APD_{50} (ms)	23.88	38.86	58.15	116.57	150.63	236.70	<0.001***
APD_{20}/APD_{90}	0.13	0.17	0.29	0.52	0.57	0.62	<0.001***
dV/dt_{max} (mV/ms)	92.56	122.20	156.04	81.93	90.72	106.91	0.005***
APA (mV)	100.72	109.91	117.18	109.55	119.43	126.13	0.011*

Supplementary Table 6. Statistical analysis of electrical biomarkers. Median and interquartile range (25th, 50th, 75th percentiles) of electrical parameters after cell clusterization by unsupervised learning (K-means algorithm; see methods). A *p*-value < 0.05 was considered significant, n = 46 cells deriving from 4 independent differentiations (E_{diast}, diastolic membrane potential; E_{diast} with DC, diastolic membrane potential with Dynamic Clamp; $APD_{90,}$ APD_{50} $_{and}$ APD_{20}; action potential duration at 90, 50 and 20% of repolarization; dV/dt_{max}, maximum AP upstroke velocity; APA, AP amplitude.)

Table 7

**K-means model**	**Prediction coefficients (k)** **Principal Component 1**	**Prediction coefficients (k)** **Principal Component 2**
C_m (pF)	0.110	0.150
E_{diast} (mV)	0.007	0.539
E_{diast} with DC (mV)	0.237	0.409
APD_{90} (ms)	0.439	0.120
APD_{20} (ms)	0.474	0.081
APD_{50} (ms)	0.465	0.086
APD_{20}/APD_{90}	0.440	0.013
dV/dt_{max} (mV/ms)	0.184	0.493
APA (mV)	0.264	0.497

Supplementary Table 7. Prediction performance of electrical parameters included in the principal component analysis. The table reports absolute values for prediction coefficients from PCA; a higher value corresponds to a higher discrimination weight for each electrical parameter included in principal component 1 and principal component 2.

Table 8

Parameters	AUC	95% CI		Asymptotical Significance	Performance		
		Lower limit	Upper limit		Cut-off	Sensitivity	Specificity
C_m (pF)	0.665	0.497	0.833	0.062	< 17.5	50.0	89.3
E_{diast} (mV)	0.621	0.450	0.792	0.170	> -27.5	66.7	60.7
E_{diast} with DC	0.732	0.585	0.880	**0.008**	< -83.4	83.3	64.3
APD_{90} (ms)	0.960	0.908	1,000	**<0.001**	> 92.1	100.0	85.7
APD_{20} (ms)	1.000	1.000	1.000	**<0.001**	> 42.5	100.0	100.0
APD_{50} (ms)	0.984	0.957	1.000	**<0.001**	> 72.9	100.0	92.9
APD_{20}/APD_{90}	0.996	0.985	1.000	**<0.001**	> 0.44	100.0	96.4
dV/dt_{max} (mV/ms)	0.746	0.604	0.604	**0.005**	< 95.1	66.7	75.0
APA (mV)	0.724	0.578	0.87	**0.011**	> 117.5	61.1	75.0

Supplementary Table 8. Performance of statistical clusterization analysis. The diagnostic performance of single electrical parameters to discriminate cells according to unsupervised clusterization (cluster 1 *vs.* cluster 2) was evaluated by analysis of ROC curves. The area under the curve (AUC) is reported together with the 95% confidence interval (CI; lower limit and upper limit). The asymptotical significance is referred to the referral line; a *p*-value < 0.05 was considered significant and showed in bold. The cut-off correspondent to the maximum accuracy was derived according to the Younden index. C_m, cell capacitance; E_{diast}, diastolic membrane potential; E_{diast} with DC, diastolic membrane potential with Dynamic Clamp; APD_{90}, APD_{50} and APD_{20}; action potential duration at 90, 50 and 20% of repolarization; dV/dt_{max}, maximum AP upstroke velocity; APA, AP amplitude.

Table 9

**Regression models**	**OR**	**95% CI**		***P*-value**
		**Lower limit**	**Upper limit**	
C_m (pF)	0.977	0.926	1.030	0.388
E_{diast} (mV)	0.967	0.918	1.019	0.209
E_{diast} with DC	0.755	0.618	0.922	**0.006**
APD_{90} (ms)	1.037	1.014	1.061	**0.002**
APD_{20} (ms)	1.225	1.065	1.410	**0.004**
APD_{50} (ms)	1.077	1.024	1.134	**0.004**
APD_{20}/APD_{90}	1.768	1.372	4.299	**0.002**
dV/dt_{max} (mV/ms)	0.959	0.931	0.987	**0.005**
APA (mV)	1.059	1.002	1.118	**0.042**

Supplementary Table 9. Univariate regression models. Univariate logistic regression showing associations between electrical parameters and cell phenotype (cluster 1 vs. cluster 2). Odds ratios (ORs) are reported with 95% confidence interval (95% CI); an OR greater than 1 is associated with an increased likelihood to belong to cluster 2; an OR less than 1 is associated with a decreased likelihood. A $p<0.05$ was considered significant and shown in bold.

Table 10

**Pearson' R**<br>***P*-value**	C_m (pF)	E_{diast} (mV)	E_{diast} with DC (mV)	APD_{90} (ms)	APD_{20} (ms)	APD_{50} (ms)	APD_{20}/<br>APD_{90}	dV/dt_{max} (mV/ms)	APA (mV)
C_m (pF)	**1.000**<br>**<0.001**	-0.093<br>0.539	0.041<br>0.786	-0.166<br>0.271	-0.175<br>0.245	-0.172<br>0.254	-0.074<br>0.625	0.187<br>0.214	-0.155<br>0.304
E_{diast} (mV)		**1.000**<br>**<0.001**	0.254<br>0.088	0.079<br>0.599	0.042<br>0.781	0.046<br>0.763	-0.093<br>0.539	-0.226<br>0.131	-0.194<br>0.197
E_{diast} with DC (mV)			**1.000**<br>**<0.001**	-0.133<br>0.378	**-.346***<br>**0.018**	-0.266<br>0.074	**-.567****<br>**0.000**	0.132<br>0.381	**-.513****<br>**0.000**
APD_{90} (ms)				**1.000**<br>**<0.001**	**.943****<br>**0.000**	**.983****<br>**0.000**	**.700****<br>**0.000**	-0.233<br>0.120	**.409****<br>**0.005**
APD_{20} (ms)					**1.000**<br>**<0.001**	**.983****<br>**0.000**	**.845****<br>**0.000**	**-.346***<br>**0.018**	**.430****<br>**0.003**
APD_{50} (ms)						**1.000**<br>**<0.001**	**.788****<br>**0.000**	-0.289<br>0.051	**.436****<br>**0.002**
APD_{20}/APD90							**1.000**<br>**<0.001**	**-.490****<br>**0.001**	**.401****<br>**0.006**
dV/dt_{max} (mV/ms)								**1.000**<br>**<0.001**	0.289<br>0.051
APA (mV)									**1.000**<br>**<0.001**

Supplementary Table 10. Correlations. Correlations between electrical parameters was evaluated by Pearson's R test. Pearson's R coefficient (above) and p-values (below) are reported for each comparison. A p<0.05 was considered significant and shown in bold.

Table 11

4-AP effect (delta%)	Atrial-like (n=10)	Ventricular-like (n=9)
APD_{90}	19 ± 6.7	4.6 ± 1.9
APD_{50}	36.4 ± 11.9 *	6.7 ± 2.7
APD_{20}	56.8 ± 20.2 *	6.6 ± 3
APD_{20}/APD_{90}	0.3 ± 0.1 *	0.7 ± 0.1

Supplementary Table 11. 4-AP effect on different AP phase of based-model classified atrial- and ventricular-like cells. (n=4 Std and RA parallel differentiations each including on average 10 recorded cells; Data are presented as mean±SE). APD_{90}, APD_{50} and APD_{20}; action potential duration at 90%, 50% and 20% of repolarization**.**

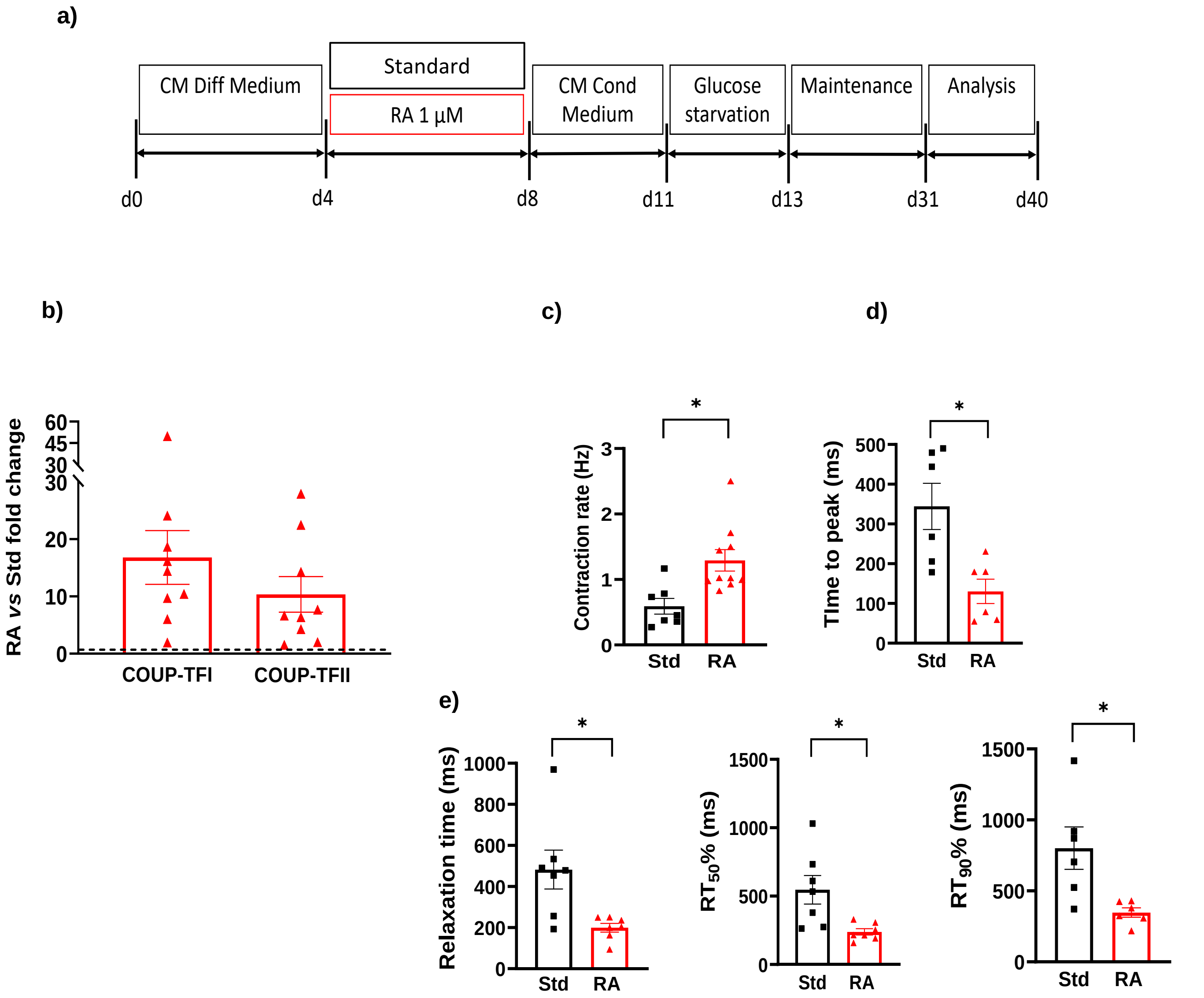

Supplementary Figure 1. (a) Timeline of parallel differentiation protocols in standard (Std) conditions and with administration of RA, to induce atrial differentiation, from day 4 to 8. Glucose starvation was applied from day 11 to 13 to purify hiPSC-CMs. **(b)** The molecular investigation of COUP-TFI and COUP-TFII was expressed as RA/St d fold change; (n = 9 technical replicates from 4 independent differentiations). **(c)** Musclemotion analysis: contraction rate (Hz); (Std n=7 and RA n=10), time to peak (ms) (Std n=6 and RA n=6), relaxation time (ms) (Std n=7 and RA n=7), RT (ms) at 50% amplitude (Std n=7 and RA n=7) and 90% of the amplitude (Std n=6 and RA n=6) from 6 independent differentiations. Data are presented as mean±SE.

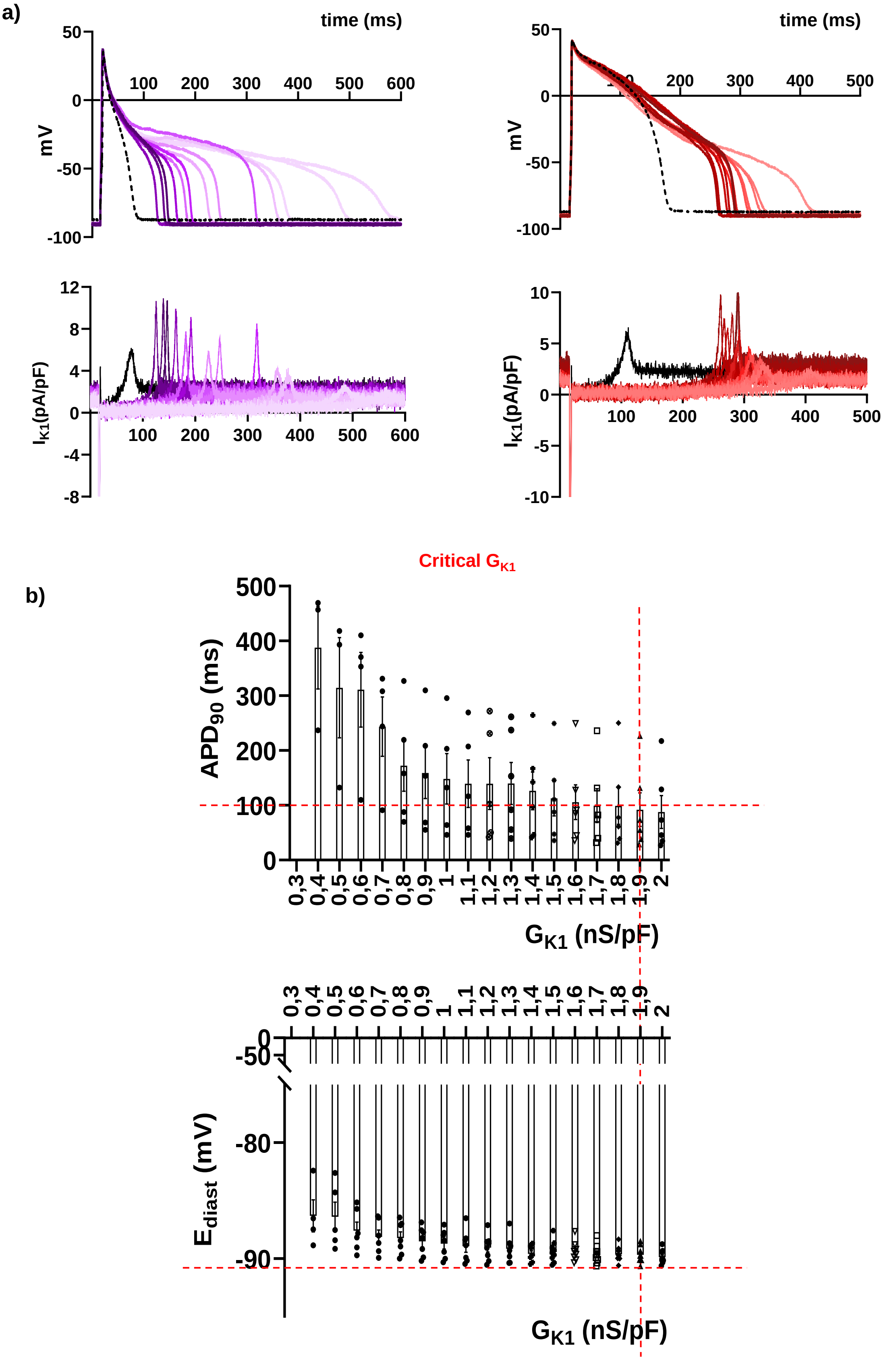

Supplementary Figure 2. G_{K1} parameter setting of I_{K1_Ventr} model. (**a**) Examples of evoked shorter (atrial-like) and longer (ventricular-like) AP profiles following progressive increase of G_{K1} ranging from 0.3 to 2 nS/µF (0.1 nS/µF per step). Light colour code for low G_{K1} values and dark colours code for high G_{K1} values. For comparison, traces recorded under I_{K1_Atr} model injection are shown in black. (**b**) APD_{90} and E_{diast} changes yielded from all cardiomyocytes (n=4-8 cells from 1 independent differentiation, depending on the cell stability at lower G_{K1}) are represented against G_{K1} values. Red dashed line identifies the critical G_{K1} value to reach stable AP parameters. (APD_{90}, AP duration measured at 90% of the repolarisation phase; E_{diast}, diastolic membrane potential; G_{K1}, I_{K1} conductance) (data are presented as mean±SE).

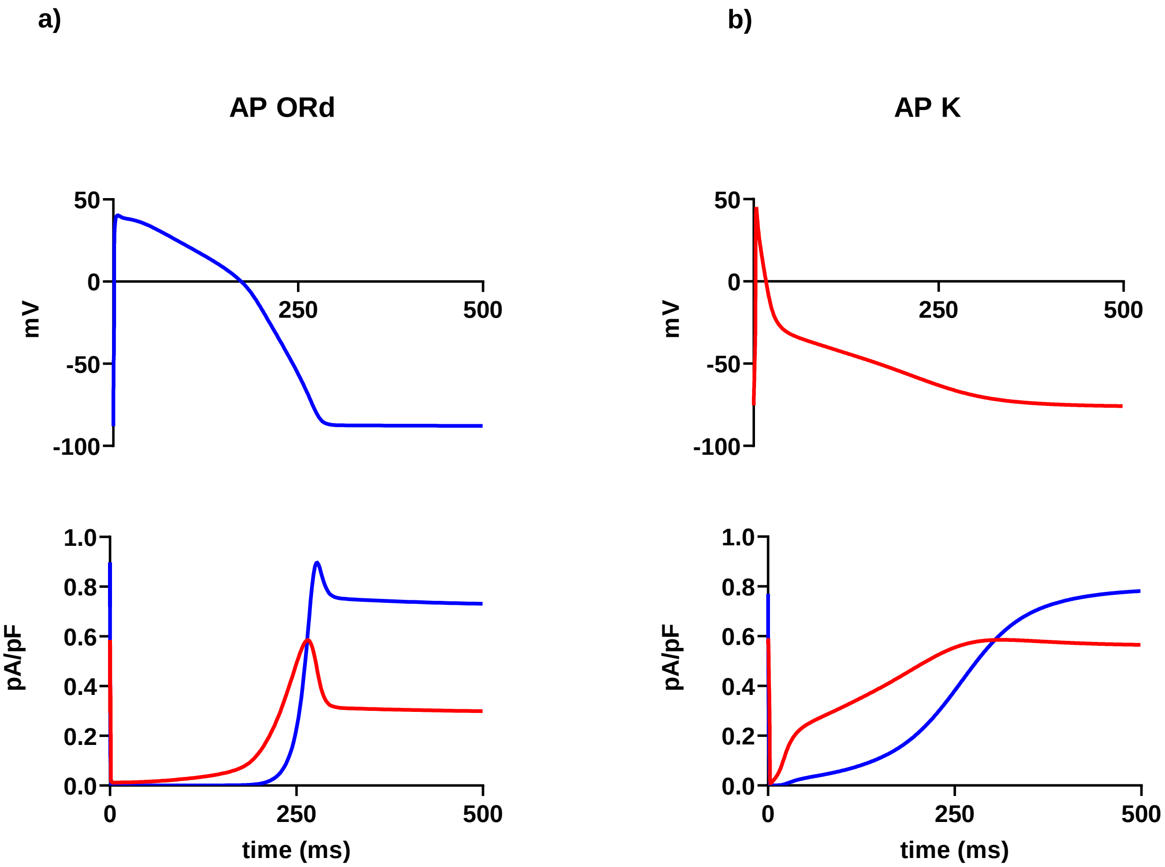

Supplementary Figure 3. Analysis of the two I_{K1} models in simulated AP-Clamp experiments using atrial and ventricular APs as voltage commands. Ventricular (left) and atrial (right) AP waveforms obtained from the ORd and the Koivumaki models, respectively, were used to clamp the I_{K1_Ventr} (blue) and I_{K1_Atr} (red) models. The current traces obtained in these simulated AP-Clamp experiments are reported in the bottom panels. It can be appreciated that I_{K1_Atr} activates earlier than the I_{K1_Ventr}. This is particularly evident with the simulated atrial-AP, leading to a substantially larger outward current all along the AP duration. At a lower extent, this is true also with the simulated ventricular-AP with I_{K1_Atr} larger than I_{K1_Ventr}, especially in the late repolarization phase.

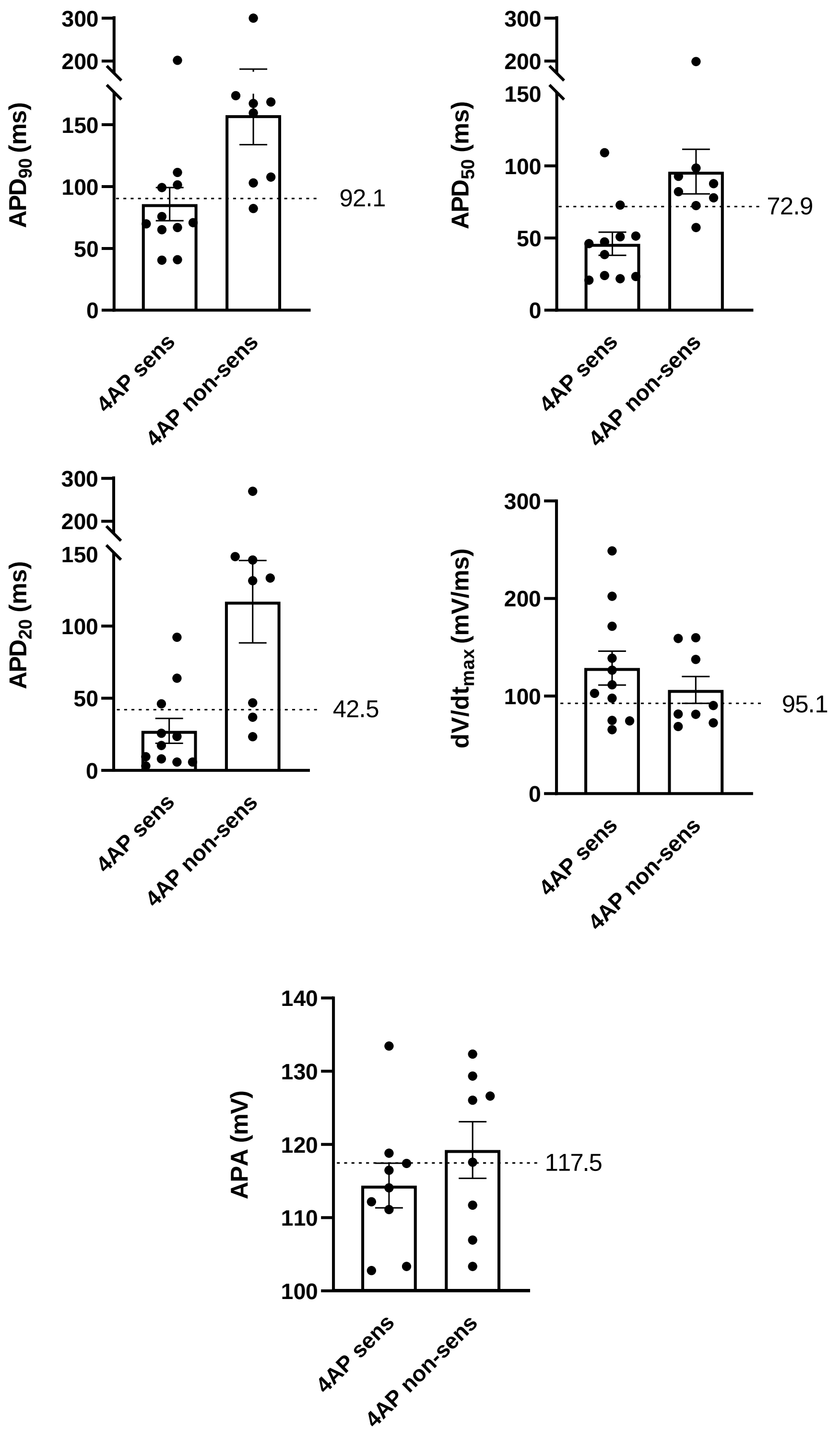

Supplementary Figure 4. Pharmacological test on electrical biomarkers. Distribution of APD_{90}, APD_{50}, APD_{20}, dV/dt_{max} and APA values for 4-AP-sensitive and 4-AP non-sensitive CMs, with respect to their critical ROC analysis-derived cut-off value (dot lines in the plots, n = 19 randomly selected cells from 4 independent differentiations; data are presented as mean±SE)
