# Supplementary material for: A dynamic clamping approach using in silico IK1 current for discrimination of chamber-specific hiPSC-derived cardiomyocytes
Source: Commun Biol. 2023 Mar 18;6:291. doi: 10.1038/s42003-023-04674-9 (PMC10024709; doi:10.1038/s42003-023-04674-9)
Supplement: Supplementary file 2 — Reporting Summary [file 42003_2023_4674_MOESM2_ESM.pdf]

nature portfolio

Corresponding author(s): COMMSBIO-22-1473B
Last updated by author(s): Jan 26, 2023

Reporting Summary

Nature Portfolio wishes to improve the reproducibility of the work that we publish. This form provides structure for consistency and transparency in reporting. For further information on Nature Portfolio policies, see our Editorial Policies and the Editorial Policy Checklist.

Statistics

For all statistical analyses, confirm that the following items are present in the figure legend, table legend, main text, or Methods section.

n/a	Confirmed	
☐	☒	The exact sample size (n) for each experimental group/condition, given as a discrete number and unit of measurement
☐	☒	A statement on whether measurements were taken from distinct samples or whether the same sample was measured repeatedly
☐	☒	The statistical test(s) used AND whether they are one- or two-sided<br>*Only common tests should be described solely by name; describe more complex techniques in the Methods section.*
☒	☐	A description of all covariates tested
☐	☒	A description of any assumptions or corrections, such as tests of normality and adjustment for multiple comparisons
☐	☒	A full description of the statistical parameters including central tendency (e.g. means) or other basic estimates (e.g. regression coefficient) AND variation (e.g. standard deviation) or associated estimates of uncertainty (e.g. confidence intervals)
☐	☒	For null hypothesis testing, the test statistic (e.g. F, t, r) with confidence intervals, effect sizes, degrees of freedom and P value noted<br>*Give P values as exact values whenever suitable.*
☒	☐	For Bayesian analysis, information on the choice of priors and Markov chain Monte Carlo settings
☒	☐	For hierarchical and complex designs, identification of the appropriate level for tests and full reporting of outcomes
☒	☐	Estimates of effect sizes (e.g. Cohen's d, Pearson's r), indicating how they were calculated

Our web collection on statistics for biologists contains articles on many of the points above.

Software and code

Policy information about availability of computer code

Data collection	To implement Dynamic Clamp, the Multiclamp 700B amplifier (Axon Instruments) was connected to a data acquisition board (DAQ, 6024E PCI, National Instruments) on a personal computer (Intel Celeron 3.20 GHz). The open-source Real-Time Experiment Interface (RTXI) was used: it is a fast and versatile real-time biological experimentation system based on Real-Time Linux. System features and custom user code were implemented as modules written in C++.
Data analysis	Multi Channel Systems, Reutlingen, Germany. Clampfit, Molecular Devices. GraphPad PRISM 8.0.2.MUSCLEMOTION ImageJ macro

For manuscripts utilizing custom algorithms or software that are central to the research but not yet described in published literature, software must be made available to editors and reviewers. We strongly encourage code deposition in a community repository (e.g. GitHub). See the Nature Portfolio guidelines for submitting code & software for further information.

Data

Policy information about availability of data

All manuscripts must include a data availability statement. This statement should provide the following information, where applicable:

- Accession codes, unique identifiers, or web links for publicly available datasets
- A description of any restrictions on data availability
- For clinical datasets or third party data, please ensure that the statement adheres to our policy

Datasets are available in github.com. Links for publicly available datasets is https://github.com/CardiovascularTheranostics/DynamicClamp.git

Human research participants

Policy information about studies involving human research participants and Sex and Gender in Research.

Reporting on sex and gender	N/A
Population characteristics	N/A
Recruitment	N/A
Ethics oversight	N/A

Note that full information on the approval of the study protocol must also be provided in the manuscript.

Field-specific reporting

Please select the one below that is the best fit for your research. If you are not sure, read the appropriate sections before making your selection.

☒ Life sciences ☐ Behavioural & social sciences ☐ Ecological, evolutionary & environmental sciences

For a reference copy of the document with all sections, see nature.com/documents/nr-reporting-summary-flat.pdf

Life sciences study design

All studies must disclose on these points even when the disclosure is negative.

Sample size	no sample size was pre-determinate. Sample Size was determined by empirical determination of the optimum sample size for the minimum number technical replicates to identify any statistically significant
Data exclusions	no data exclusion
Replication	Two clonally derived hiPSC lines from the same subject were used as cell source to obtain hiPSC-CMs using the two differentiation protocols (Std and RA). For electrophysiology experiments “n” refers to number of recorded single cell/cluster, deriving from independent differentiations. Independent experiments (differentiations) are indicated in the method and in the figure legends for each experiment.
Randomization	N/A
Blinding	N/A

Reporting for specific materials, systems and methods

We require information from authors about some types of materials, experimental systems and methods used in many studies. Here, indicate whether each material, system or method listed is relevant to your study. If you are not sure if a list item applies to your research, read the appropriate section before selecting a response.

Materials & experimental systems

n/a	Involved in the study	
☐	☒	Antibodies
☐	☒	Eukaryotic cell lines
☒	☐	Palaeontology and archaeology
☒	☐	Animals and other organisms
☒	☐	Clinical data
☒	☐	Dual use research of concern

Methods

n/a	Involved in the study	
☒	☐	ChIP-seq
☒	☐	Flow cytometry
☒	☐	MRI-based neuroimaging

Antibodies

Antibodies used

Anti-MLC2v; Proteintec 10906-1-AP, Anti-MLC2a; Synaptic System 311011. Alexa Fluor secondary antibodies (Thermo Fisher Scientific) was used for detection

Validation

Validation by WB and immunoflurescence in mouse and human heart tissue https://www.ptglab.com/products/MYL2-Antibody-10906-1-AP.htm#product-information

Eukaryotic cell lines

Policy information about cell lines and Sex and Gender in Research

Cell line source(s)

Induced pluripotent stem cells from primary fibroblast as approved by the local Ethics Committee (Comitato Etico Cantonale, Bellinzona, Switzerland; Ref. CE 2923) and performed according to the Declaration of Helsinki.

Authentication

Non of the cell lines were authenticated

Mycoplasma contamination

All the cell lines were tested for mycoplasma

Commonly misidentified lines
(See ICLAC register)

N/A
